# Fluorinated Iron(ii) clathrochelate units in metalorganic based copolymers: improved porosity, iodine uptake, and dye adsorption properties†

**DOI:** 10.1039/d1ra02357h

**Published:** 2021-04-21

**Authors:** Suchetha Shetty, Noorullah Baig, Atikur Hassan, Saleh Al-Mousawi, Neeladri Das, Bassam Alameddine

**Affiliations:** Department of Mathematics and Natural Sciences, Gulf University for Science and Technology Kuwait alameddine.b@gust.edu.kw; Functional Materials Group – CAMB, GUST Kuwait; Department of Chemistry, Indian Institute of Technology Patna Patna 801106 Bihar India; Department of Chemistry, University of Kuwait Kuwait

## Abstract

We report the synthesis of metalorganic copolymers made from the palladium catalyzed Sonogashira cross-coupling reaction between various iron(ii) clathrochelate building blocks with diethynyl–triptycene and fluorene derivatives. The target copolymers CCP1–5 were isolated in excellent yield and characterized by various instrumental analysis techniques. Interestingly, investigation of the copolymers' porosity properties discloses BET surface areas up to 337 m^2^ g^−1^ for the target compounds bearing fluorinated iron(ii) clathrochelate units CCP2,5. Moreover, the fluorinated copolymers display an outstanding uptake capacity of iodine with a maximum adsorption of 200 wt%. The target metalorganic copolymers CCP1–5 reveal very good adsorption of organic dyes, namely, methyl blue and methylene blue, from aqueous media.

## Abstract 2

We report the synthesis of metalorganic copolymers made from the palladium catalyzed Sonogashira cross-coupling reaction between various iron(ii) clathrochelate building blocks with diethynyl–triptycene and fluorene derivatives.

## Graphical abstract

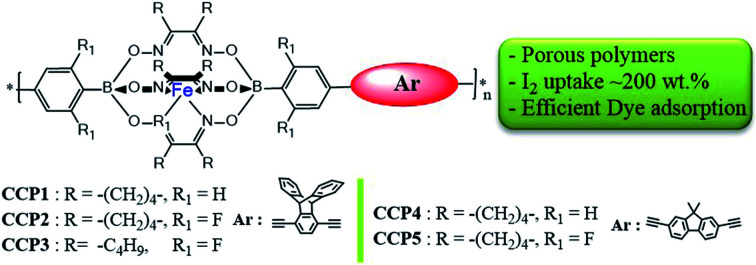

## 1. Introduction

In the past two decades, there has been growing interest in designing amorphous polymers with contorted structures that induce poor three-dimensional packing in the solid state, therefore, leading to the formation of intrinsic pores which bestow the resulting materials with prominent properties allowing for their use in various applications, namely, gas storage and separation, sensing, and catalysis.^1–7^ Unlike highly ordered materials which require complex synthesis and isolation methodologies, such as metal–organic frameworks (MOFs)^8,9^ and covalent organic frameworks (COFs),^10,11^ microporous polymer networks (MPNs),^12^ in particular, polymers of intrinsic microporosity (PIMs)^13,14^ are renowned for their versatile synthesis and utilization in energy storage technologies, such as, (i) natural gas adsorbents for vehicles, (ii) hydrogen gas tanks for fuel cells, and (iii) vessels for CO_2_ separation/sequestration from flue gas.^7,15–22^ Amid the various synthetic methodologies to prepare microporous polymers, palladium-catalyzed cross-coupling reactions, namely, Suzuki^23^ and Sonogashira^24^ offer numerous advantages, among others, allowing the preparation of target microporous materials from hands-on synthons which are either commercially available or might require few synthetic steps. In addition, the abovementioned cross-coupling reactions permit a noticeable control over the polymerization reaction, thus, yielding highly structured copolymers. On top that, Suzuki and Sonogashira cross-coupling reactions are powerful synthetic tools for the preparation of porous materials with additional properties, such as, making conjugated polymers, introducing functional groups into the macromolecules' backbones, *etc.*^1,25^ Typically, palladium complexes in combination with copper(i) salts are employed as catalysts in Sonogashira cross-coupling reactions although alternative catalytic systems were also found to be very effective.^26–28^

Iron(ii) clathrochelate is a robust metalorganic unit with a considerable internal free volume (IFV) and which can be easily synthesized and functionalized, especially when employed as a building block in cross-coupling reactions.^29–31^ Iron(ii) clathrochelate complexes were tested as biosensors,^32,33^ catalysts for hydrogen generation,^34,35^ materials for electronic transport,^36^ organogels,^37^ and to make supramolecular structures of definite shapes and sizes.^38–41^ Recently, several iron(ii) clathrochelate based polymers were reported disclosing prominent porous properties.^42–44^ In this prospect, we report herein the synthesis and characterization of five new iron(ii) clathrochelate copolymers CCP1–5 using Sonogashira cross-coupling reaction conditions. Interestingly, the target copolymers exhibit Brunauer–Emmett–Teller (BET) surface area up to 337 m^2^ g^−1^ as well as high iodine uptake and excellent adsorption of organic dyes, namely, methyl blue and methylene blue.

## 2 Experimental part

### 2.1 General

All the reactions were carried out under inert atmosphere using dry argon. All chemical reagents were used without further purification as purchased from Aldrich, Merck, and HiMedia unless otherwise specified. 1,4-Diethynyl-9,10-dihydro-9,10-[1,2]benzenoanthracene 1a, 2,7-diethynyl-9,9-dimethyl-9*H*-fluorene 1b, and CC1 were prepared according to procedures reported in the literature.^41,45,46^ The solvents, namely, DMF, diethyl ether, methanol, hexane, DCM and THF were deoxygenated by bubbling with dry argon gas for 30 minutes. Thin-layer chromatography (TLC) was performed on aluminum sheets coated with silica gel 60 F254 and revealed using a UV lamp. NMR (^1^H: 600 MHz, ^13^C: 150 MHz) spectra were recorded on Bruker BioSpin GmbH 600 MHz spectrometer using CD_2_Cl_2_ as a solvent with the chemical shifts (*δ*) given in ppm and referred to tetramethylsilane (TMS). FT-IR spectra were recorded on Agilent Cary 630 FTIR instrument. UV-Vis spectra were recorded on Shimadzu UV1800 spectrophotometer. Brunauer–Emmett–Teller (BET) surface area and porosity measurements were evaluated using a Surface Area and Pore Size Analyzer (Gemini-V, Micromeritics, USA) at the boiling point of liquid nitrogen (−196 °C). Samples were degassed in VacuPrep 061 sample degassing system at a temperature of 105 °C for overnight, before the experiments. Surface areas (SBET) were calculated using the Brunauer–Emmet–Teller (BET) model of isotherms, and the adsorption of N_2_ at small relative pressures. Total pore volume (*V*_t_) was determined from the specific adsorption of N_2_ at a *p*/*p*^0^ = 0.99. The *t*-plot method was used to estimate micropore volume (*V*_mic_) and external surface area (*S*_ext_). Quantachrome Autosorb iQ2 analyzer was used to collect gas adsorption data. In a typical gas uptake setup, samples (120–150 mg) were loaded in a 9 mm cell and were subjected to degassing at 120 °C for 5–8 h by attaching to the degassing unit. The cells with the degassed materials were refilled with helium gas and weighed accurately. Subsequently, cells were reattached to the analysis unit of the instrument for measurements. Various temperatures of the analysis unit sample cell were maintained using KGW isotherm bath that was filled with liquid N_2_ (77 K), or using a temperature-controlled bath (298 K and 273 K). Polymers molecular weights were recorded on Agilent 1260 infinity II gel permeation chromatograph (GPC) against with a refractive index (RI) detector at room temperature and two columns (PL mixed-C) which are calibrated twelve monodisperse polystyrene (PS) standards with THF employed as eluent at a flow rate of 1.0 mL min^−1^ UV-Vis spectra were recorded on Shimadzu UV1800 spectrophotometer. X-ray Photoelectron spectroscopy (XPS) data were recorded with a Thermo ESCALAB 250 Xi using a monochromatic Al Kα-radiation source (1486.6 eV) with a spot size of 850 μm. Spectra acquisition and processing were carried out using the software Thermo Advantage Version 4.87. The base pressure in the XPS analysis chamber was in the range 10^−10^ to 10^−9^ torr. The analyzer was operated with pass energy of 20 eV, dwell time of 50 min and with a step size of 0.1 eV.

Electron impact high-resolution mass spectra (EI-HRMS) were recorded on a Thermo (DFS) with a standard PFK (perfluorokerosene) as lock mass. The analyzed data is converted to accurate mass employing X-Calibur accurate mass calculation software.

### 2.2 Synthesis

#### 2.2.1 Synthesis of CC2 (procedure A)

A Schlenk tube was charged with nioxime (400 mg, 2.74 mmol, 3 eq.), 4-bromo-2,6-difluorophenylboronic acid (500 mg, 2.11 mmol, 2 eq.) and anhydrous FeCl_2_ (115 mg, 0.91 mmol) in MeOH (20 mL) and the mixture was refluxed for 3 h under argon. The reaction mixture was allowed to cool to RT, and the resulting precipitate was isolated by filtration, washed with MeOH, diethyl ether, and dried under vacuum to yield a red solid (700 mg, 88%); ^1^H-NMR (600 MHz, CD_2_Cl_2_, ppm): *δ* 7.05–7.04 (d, *J* = 6 Hz, 4H, ArH), 2.92 (s, 12H, CH_2_), 1.83 (s, 12H, CH_2_); ^13^C-NMR (150 MHz, CD_2_Cl_2_, ppm): *δ* 167.98, 166.31, 152.96, 122.24,115.69, 26.75, 22.11; HRMS: *m*/*z* calculated for (M˙^+^) C_30_H_28_B_2_Br_2_F_4_FeN_6_O_6_ 881.9883 found 881.9885.

#### 2.2.2 Synthesis of CC3

CC3 was prepared following procedure A with: butyl dioxime (165 mg, 0.82 mmol, 3 eq.), 4-bromo-2,6-difluorophenylboronic acid (150 mg, 0.633 mmol, 2 eq.) and anhydrous FeCl_2_ (36 mg, 0.29 mmol) in MeOH (10 mL). Red solid (290 mg, 96%); ^1^H-NMR (600 MHz, CD_2_Cl_2_, ppm): *δ* 7.03–7.02 (d, *J* = 6 Hz, 4H, ArH), 2.82–2.79 (t, *J* = 6 Hz, 12H, CH_2_), 1.56–1.53 (m, 12H, CH_2_), 1.34 1.30 (m, 12H, CH_2_), 0.89–0.86 (t, *J* = 6 Hz, 18H, CH_3_); ^13^C-NMR (150 MHz, CD_2_Cl_2_, ppm): *δ* 168.15, 166.48, 157.94, 121.92, 115.60, 29.66, 27.69, 23.00 14.06; HRMS: *m*/*z* calculated for (M˙^+^) C_42_H_58_B_2_Br_2_F_4_FeN_6_O_6_ 1056.2230 found 1056.2236.

#### 2.2.3 Synthesis of copolymer CCP1 (procedure B)

A Schlenk tube was charged with a mixture of DMF and iPr_2_NH (1 : 1, 10 mL) which was bubbled with argon for 30 min. Before the addition of CC1 (200 mg, 0.25 mmol, 1 eq.) and 1a ( 75 mg, 0.25 mmol, 1 eq.). The mixture was purged with argon for 10 min. Before adding CuI (4 mg, 0.02 mmol, 8 mol%) and Pd(PPh_3_)_4_ (23 mg, 0.02 mmol, 8 mol%) under a positive stream of argon. The reaction was stirred for 48 h at 110 °C and the precipitate was isolated by filtration while hot and washed with DMF (50 mL), water (50 mL), THF (50 mL), DCM (50 mL), acetone (50 mL), methanol (50 mL) and diethyl ether (50 mL) to yield a red solid (211 mg, 90%); FTIR (cm^−1^): 2955 (aliphatic –C–H stretch.), 2201 (C≡C stretch.), 1664 (C=N stretch.), 1550 (N–O stretch.), 1431 (aliphatic –C–H bend.), and 954 (aromatic C=C bend.); UV-vis: (THF, 10^−8^ M), *λ*_max_ [nm] = 329 and 450.

#### 2.2.4 Synthesis of copolymer CCP2

CCP2 was prepared following procedure B with: CC2 (200 mg, 0.23 mmol, 1 eq.), 1a (69 mg, 0.23 mmol, 1 eq.), Pd(PPh_3_)_4_ (20 mg, 0.018 mmol, 8 mol%) and CuI (3 mg, 0.018 mmol, 8 mol%) in 9 mL of a 1 : 1 degassed mixture of DMF and iPr_2_NH. Red solid (200 mg, 87%). FTIR (cm^−1^): 2944 (aliphatic –C–H stretch.), 2201 (C≡C stretch.), 1618 (C=N stretch.), 1545 (N–O stretch.), 1458 (aliphatic –C–H bend.), 1395 (C–F stretch.), and 964 (aromatic C=C bend.); UV-vis: (THF, 10^−8^ M), *λ*_max_ [nm] = 329 and 450.

#### 2.2.5 Synthesis of copolymer CCP3

CCP3 was prepared following procedure B with: CC3 (250 mg, 0.24 mmol, 1 eq.), 1a (72 mg, 0.24 mmol, 1 eq.), Pd(PPh_3_)_4_ (22 mg, 0.019 mmol, 8 mol%) and CuI (4 mg, 0.019 mmol, 8 mol%) in 10 mL of a 1 : 1 degassed mixture of DMF and iPr_2_NH. The solvent was evaporated under reduced pressure and the resulting residue was extracted with ethyl acetate from an aqueous solution of 10% LiCl (100 mL). The organic layer was washed with deionized water (100 mL × 3), concentrated, and the product was precipitated by adding hexane. The precipitate was isolated by filtration under reduced pressure and washed exhaustively with hexane. Red solid (261 mg, 92%);^1^H-NMR (600 MHz, CD_2_Cl_2_, ppm): *δ* 7.60–7.53 (m, 4H, ArH), 7.31–7.09 (m, 10H, ArH), 6.03 (br, 2H, triptycene CH), 2.87 (br, 12H, butyl-CH_2_), 1.60 (br, 12H, butyl-CH_2_), 1.38 (br, 12H, butyl-CH_2_), 0.93 (br, 18H, butyl-CH_3_); ^13^C-NMR (150 MHz, CD_2_Cl_2_, ppm): *δ* 167.96, 166.32, 157.94, 148.21, 145.02, 129.68, 128.68, 126.23, 124.63, 115.58, 115.35, 114.98, 114.76, 52.62, 29.70, 27.69, 23.03, 14.11; FTIR (cm^−1^): 2958 (aliphatic –C–H stretch.), 2210 (C≡C stretch.), 1618 (C=N stretch.), 1544 (N–O stretch.), 1458 (aliphatic –C–H bend.), 1400 (C–F stretch.), and 716 (aromatic C=C bend.); GPC (THF): *M*_w_ (g mol^−1^): 14 106, *M*_n_ (g mol^−1^): 7388, *Đ*: 1.9; UV-vis: (THF, 10^−8^ M), *λ*_max_ [nm] = 329 and 450.

#### 2.2.6 Synthesis of copolymer CCP4

CCP4 was prepared following procedure B with: CC1 (300 mg, 0.37 mmol, 1 eq.), 1b (90 mg, 0.37 mmol, 1 eq.), Pd(PPh_3_)_4_ (34 mg, 0.03 mmol, 8 mol%) and CuI (5 mg, 0.03 mmol, 8 mol%) in 15 mL of a 1 : 1 degassed mixture of DMF and iPr_2_NH. Red solid (301 mg, 90%); FTIR (cm^−1^): 2955 (aliphatic –C–H stretch.), 2201 (C≡C stretch.), 1602 (C=N stretch.), 1492 (N–O stretch.), 1434 (aliphatic –C–H bend.), and 960 (aromatic C=C bend.); UV-vis: (THF, 10^−8^ M), *λ*_max_ [nm] = 354, 371, 400 and 450.

#### 2.2.7 Synthesis of copolymer CCP5

CCP5 was prepared following procedure B with: CC2 (180 mg, 0.21 mmol, 1 eq.), 1b (50 mg, 0.21 mmol, 1 eq.), Pd(PPh_3_)_4_ (19 mg, 0.017 mmol, 8 mol%) and CuI (3 mg, 0.017 mmol, 8 mol%) in 8 mL of a 1 : 1 degassed mixture of DMF and iPr_2_NH. Red solid (188 mg, 95%); FTIR (cm^−1^): 2942 (aliphatic –C–H stretch.), 2207 (C≡C stretch.), 1618 (C=N stretch.), 1542 (N–O stretch.), 1436 (aliphatic –C–H bend.), 1396 (C–F stretch.), and 959 (aromatic C=C bend.); UV-vis: (THF, 10^−8^ M), *λ*_max_ [nm] = 354, 371, 400 and 450.

## 3. Results and discussion

**Scheme 1:**
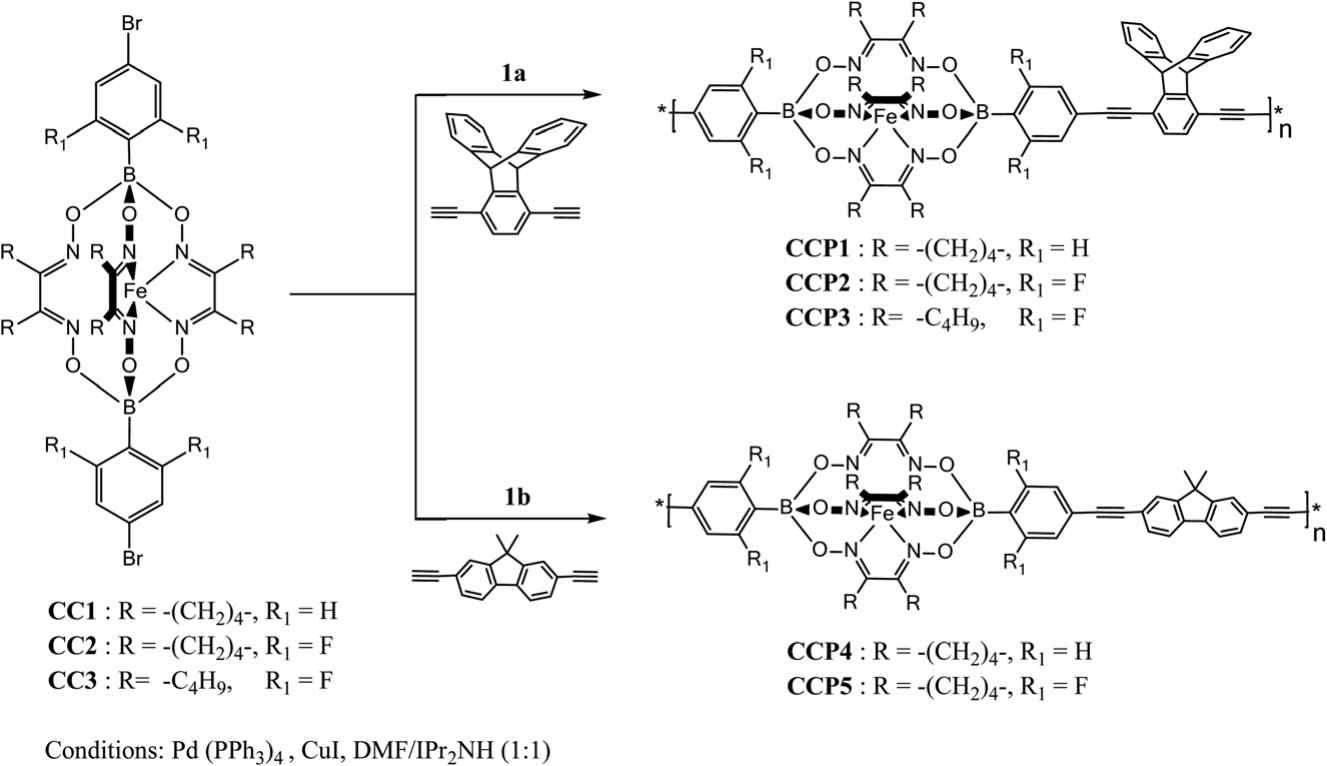
Synthesis of copolymers CCP1–5.

**Fig. 1:**
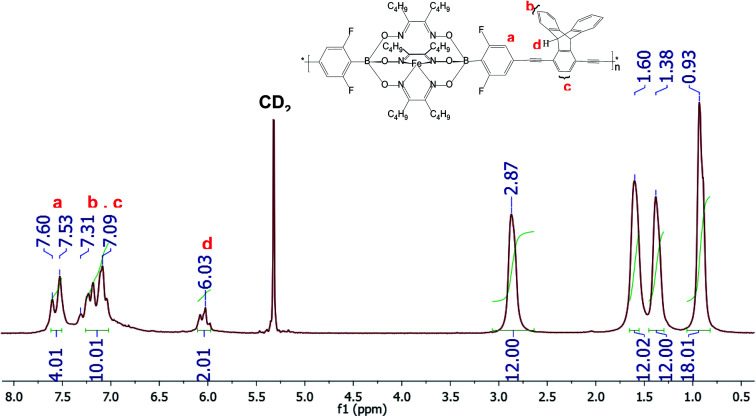
^1^H-NMR spectrum of CCP3 recorded in CD_2_Cl_2_.

**Fig. 2:**
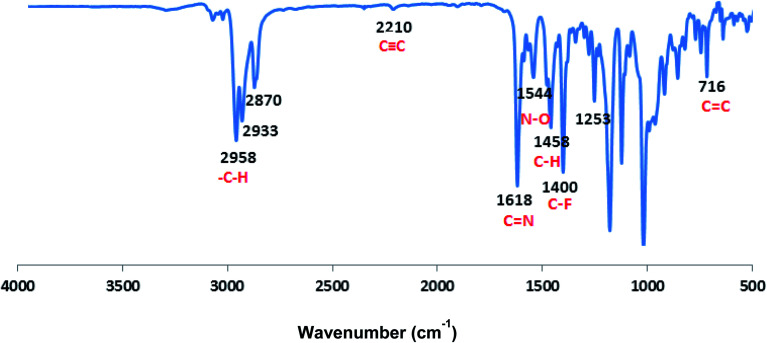
FTIR spectrum of CCP3.

Scheme 1 depicts the synthetic strategy which was devised to prepare the target clathrochelate copolymers CCP1–5*via* a typical Sonogashira cross-coupling reaction condition using the dibrominated iron(ii) clathrochelate synthons CC1–3 with each of 1,4-diethynyl triptycene 1a and 2,7-diethynyl-9,9-dimethyl fluorene 1b. The desired copolymers CCP1–5 were isolated after 2 days of reaction at 110 °C by simple filtration while hot followed by exhaustive sequential washings of the precipitates with hot solvents, thus, affording the desired target materials in excellent yields (87–95%). The copolymers whose clathrochelate units bear cyclohexyl side groups (*i.e.*CCP1,2,4,5) were found to be highly insoluble in common organic solvents. Consequently, the hitherto mentioned four copolymers were characterized using FTIR and XPS spectroscopy (Fig. S8–S15 in the ESI† file). Nevertheless, copolymer CCP3, which contains clathrochelate units with the more flexible butyl side groups, was found to be soluble in most common organic solvents (*e.g.* DCM, THF, chloroform, acetone, and DMF), therefore, allowing for its thorough structural analysis by ^1^H- and ^13^C- NMR, FTIR, and XPS spectroscopy (Fig. 1, 2, 4 and S5 in the ESI† file).

Fig. 1 below portrays the ^1^H-NMR spectrum of CCP3 in CD_2_Cl_2_ where the chemical shifts ranging from 7.60 ppm to 7.53 ppm are attributed to the four aromatic protons of the difluorobenzene boronate groups of the iron(ii) clathrochelate unit whereas the four peaks observed at 2.87 ppm, 1.6 ppm, 1.38 ppm, and 0.93 ppm are assigned to the butyl side chains. Furthermore, the characteristic chemical shift of the sp^3^ protons in the triptycene unit is detected at 6.03 ppm whereas the aromatic protons of this latter are identified in the range of 7.31–7.09 ppm. In addition, ^13^C-NMR spectrum of CCP3 displays all the characteristic chemical shifts, which further confirms its successful formation in high purity (Fig. S5 in the ESI† file).

The FT-IR absorption spectrum shown in Fig. 2 divulges all the desired peaks of target copolymer CCP3 which clearly confirms its formation. Hence, the peaks observed at 1618 cm^−1^ and 1400 cm^−1^ are attributed to the characteristic stretching vibrations of C=N and C–F, respectively. Similarly, the peaks detected at 2958 cm^−1^ and 1544 cm^−1^ are assigned to the stretching vibrations of the aliphatic C–H and N–O groups, respectively. Moreover, the absorption peaks identified at 1458 cm^−1^ and 716 cm^−1^ correspond to the bending vibrations of the aliphatic C–H and aromatic C=C, respectively. It is noteworthy that the C≡C stretching vibrations is also spotted at 2210 cm^−1^. Similarly, all the other target copolymers disclose the characteristic peaks, thus, confirming their successful synthesis (Fig. S8–S11 in the ESI† file).

**Fig. 3:**
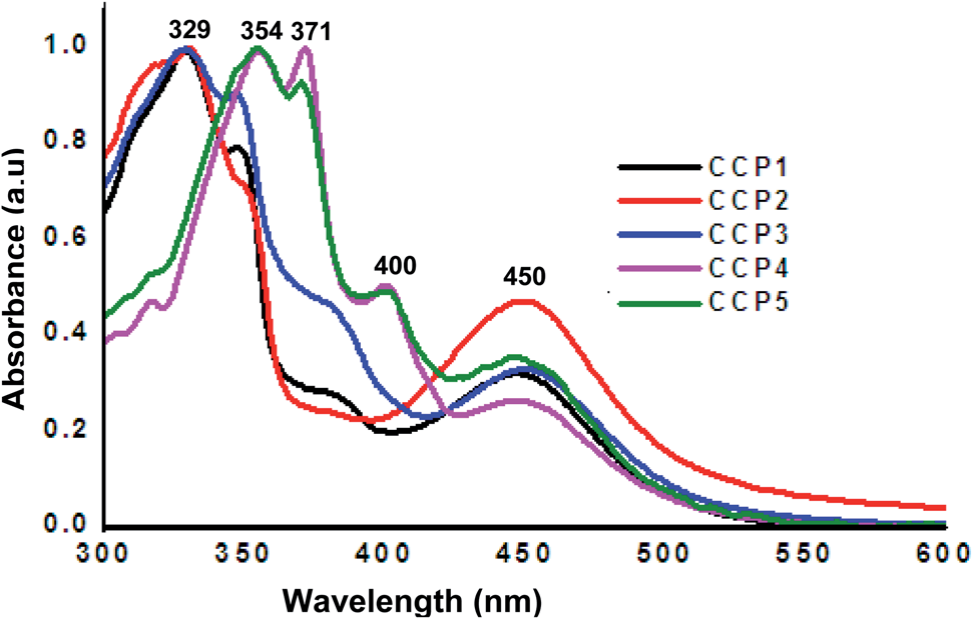
Normalized UV-Vis absorption (*C*_M_ = 10^−8^ M in THF) spectra of CCP1–5.

The photophysical properties of CCP1–5 were investigated by means of UV-Vis absorption spectroscopy using THF as a solvent (Fig. 3). Iron(ii) clathrochelate copolymers with triptycene units CCP1–3 display analogous absorption bands with two peaks maxima at ∼329 nm and 450 nm, where the former can be attributed to the absorption of triptycene^47^ while the latter corresponds to the clathrochelate moiety.^47^ On the other hand, clathrochelate copolymers with fluorene units CCP4,5 display three main absorption bands maxima, two of which are attributed to fluorene at ∼354 nm and 371 nm,^48^ whereas the one detected at 450 nm corresponds to the clathrochelate unit.

**Fig. 4:**
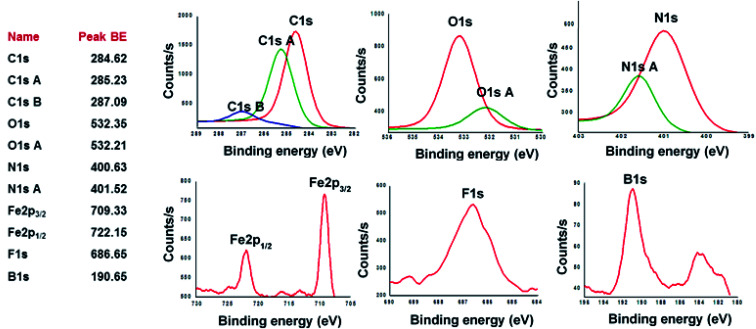
High-resolution XPS spectra of C1s, O1s, N1s, Fe2p, F1s and B1s of CCP3.

X-ray photoelectron spectroscopy (XPS) was utilized to analyze the elemental composition of copolymers CCP1–5. XPS survey-scan spectrum of CCP3, shown in Fig. 4 below, confirms the presence of all the constituting elements *i.e.* carbon, oxygen, nitrogen, iron, fluorine, and boron.^49^ C1s peak of CCP3 can be fitted into two main binding energy values at ∼284.62 eV and 285.23 eV where the former is assigned to the aromatic carbon groups (C=C) whereas the latter corresponds to that of imine carbons (C=N). The binding energy for oxygen detected at ∼532.3 eV relates to a hydrocarbon bonded to boron and nitrogen. On the other hand, N1s spectrum exhibits two peaks at 400.63 eV and 401.52 eV, which correspond to carbon–nitrogen (C–N) and nitrogen–oxygen (N–O), respectively. F1s core-level spectrum was detected at 685.65 eV, thus, indicating the presence of C–F.^50^ B1s core-level spectrum was detected at 190.65 eV, which clearly divulges the presence of boron oxide (B–O) usually observed above 190 eV.^51^Fig. 4 also reveals the XPS peak for Fe2p with binding energy values of 709.33 eV and 722.15 eV, which are attributed to Fe(ii)–N compounds.^52^ It is noteworthy that the other target copolymers CCP2–5 portray similar XPS binding energy values, which undoubtedly confirm their structures (Fig. S12–S15 in the ESI† file).

**Fig. 5:**
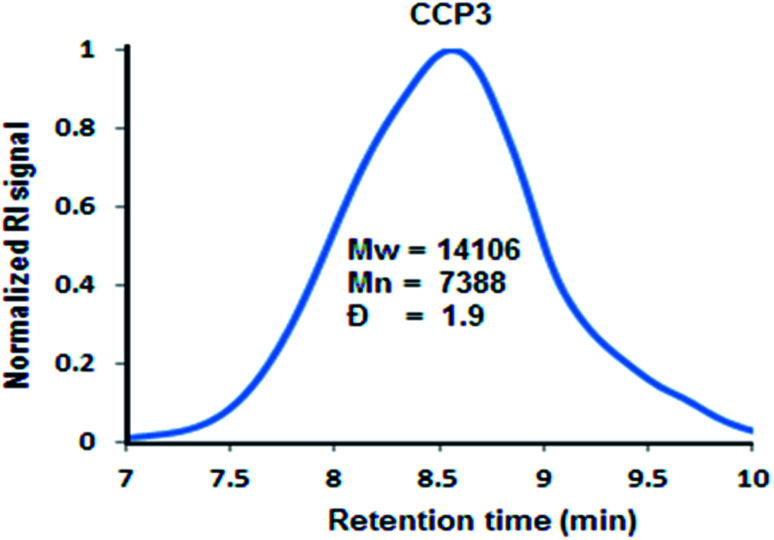
Normalized GPC chromatograph of CCP3.

As it could be noticed from Fig. 5, the relatively good solubility of clathrochelate copolymer CCP3 in common organic solvents has allowed for the determination of its molar weight by gel permeation chromatography (GPC), revealing a weight average molar mass *M*_w_ of ∼14 kDa and number average molar mass Mn of ∼7 kDa, thus, showing a polydispersity index (PDI = *M*_w_/*M*_n_) of ∼1.9. Nevertheless, the scarce solubility of the triptycene-containing copolymers CCP1,2 and those bearing dimethyl fluorene units CCP4,5 in common organic solvents prevented recording their GPC chromatograms.

## 4. Surface area and porosity analysis

**Fig. 6:**
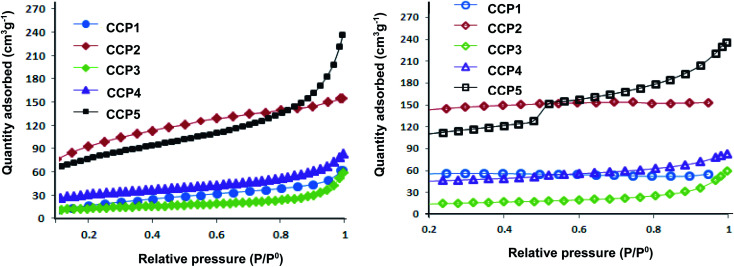
Nitrogen adsorption (left) and desorption isotherms (right) of CCP1–5 measured at 77 K.

The surface areas and porous properties of copolymers CCP1–5 were investigated by carrying out N_2_ adsorption experiments at 77 K and low relative pressure (Fig. 6). Table 1 below summarizes the Brunauer–Emmett–Teller (BET) surface areas and pore volumes derived from the nitrogen sorption isotherms. Unsurprisingly, CCP3 divulges the lowest BET surface area (∼45 m^2^ g^−1^) which can be explained by the presence of the flexible butyl side chains which block the intrinsic pores.^44^ The clathrochelate copolymers bearing triptycene CCP1 and dimethyl fluorene CCP4 units portray surface areas of ∼79 m^2^ g^−1^ and 111 m^2^ g^−1^CCP1,3, respectively. Interestingly, the replacement of the two hydrogen atoms located at the *ortho* positions with respect to boron with the bulkier and more electronegative fluorine atoms in the iron(ii) clathrochelate moiety of both copolymers *i.e.* the one with dimethyl fluorene units CCP5 and that with triptycene derivatives CCP2 resulted in a noticeable increase of the surface areas of 279 m^2^ g^−1^, for the former and 337 m^2^ g^−1^, for the latter. All five target copolymers were found to have pore volumes ranging between 0.039 cm^3^ g^−1^ and 0.222 cm^3^ g^−1^ (Table 1).

**Table 1:** Summary of the microporosity properties of polymers CCP1–5

Entry	Polymer	BET surface area (m^2^ g^−1^)	Pore volume (cm^3^ g^−1^)
1	CCP1	79	0.086
2	CCP2	337	0.214
3	CCP3	45	0.039
4	CCP4	111	0.085
5	CCP5	279	0.222

## 5. Iodine uptake study

**Fig. 7:**
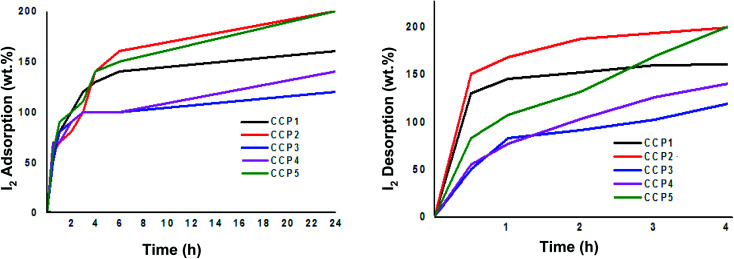
Wt% iodine adsorption (left) and desorption (right) graphs of CCP1–5.

Copolymers CCP1–5 were explored as potential iodine adsorbents using a standard gravimetric analysis protocol reported in the literature.^53,54^ The iodine uptake tests were conducted by placing a 10 mg sample for each of copolymers CCP1–5 in an open glass vial, which was in turn put inside a sealed glass vessel that contained excess solid iodine at 80 °C under atmospheric pressure and the gravimetric analysis was subsequently recorded at different time intervals (Fig. 7). Evidently, the fluorinated iron (ii) clathrochelate copolymers which revealed superior BET surface areas, *i.e.*CCP2 and CCP5, display the highest iodine uptake reaching a maximum of 200 wt%. It is noteworthy that iodine adsorption of CCP2 reached ∼160 wt% after 6 hours of exposure while that of CCP5 attained 150 wt% for the same period time. On the other hand, the maximum uptake capacities of copolymers CCP1 and CCP4 were found to reach 160 wt% and 140 wt%, respectively, after 24 hours of exposure to I_2_ (Table 2). The 200 wt% uptake for CCP2 and CCP5 to iodine is promising because of the several advantages these copolymers present, namely, their versatile synthesis from commercially available synthons and the ease of their isolation, especially when compared to the polymer networks reported in the literature which require intricate synthetic and/or purification steps and whose iodine adsorption values are either lower or approximately equal to the ones presented herein.^53–65^

**Table 2:** Summary of iodine adsorption and desorption of copolymers CCP1–5

Entry	Time (h)	Wt% I_2_ adsorption after 24 h	Wt% I_2_ desorption after 4 h
1	CCP1	160	160
2	CCP2	200	199
3	CCP3	120	119
4	CCP4	140	140
5	CCP5	200	200

Iodine-containing copolymers CCP1–5 (I_2_@CCP1–5) underwent desorption by simple heating in air at 120 °C. The complete wt% of iodine released from I_2_@CCP1–5 samples was recorded at different time intervals (Fig. 7 and Table 2). In addition, reusability tests of the copolymers were carried out using CCP2 as a model sample because it disclosed the maximum uptake capacity for iodine. Hence, a sample of CCP2 fully loaded with iodine *i.e.* I_2_@CCP2 was heated at 120 °C for 24 hours, to ensure the complete release of the adsorbate. The reactivated sample CCP2 was subsequently exposed to iodine vapors and its uptake values were recorded gravimetrically using the procedure described above, which revealed similar results to a freshly prepared copolymer after three iodine adsorption–desorption cycles.

**Fig. 8:**
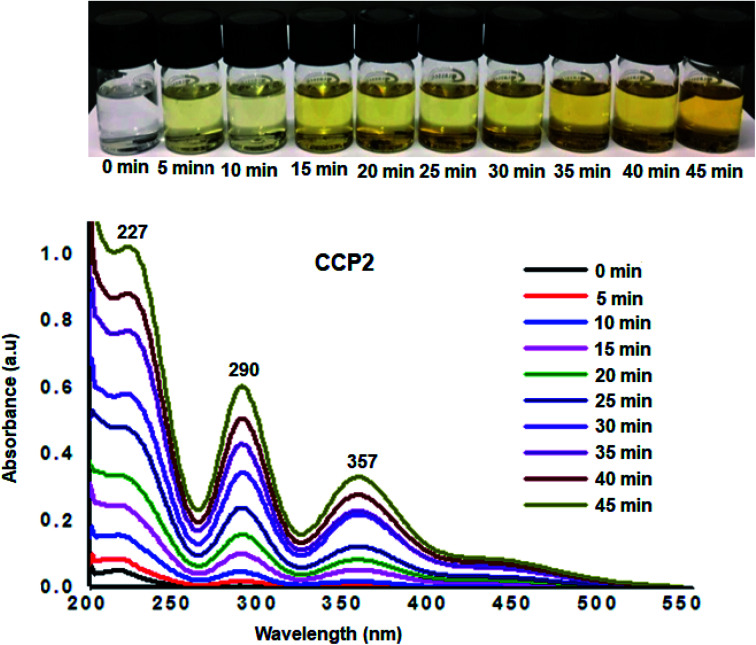
UV-Vis absorption spectra upon immersion of I_2_@CCP2 in ethanol. Inset: photos of the solutions showing the color change upon immersion in ethanol.

Additional desorption tests were investigated by immersing fully loaded samples of I_2_@CCP1–5 in ethanol, where the latter is an excellent solvent for iodine. The release of iodine from I_2_@CCP1–5 samples suspended in ethanol was analyzed by recording the UV-visible absorbance spectra of ethanol at different time intervals (Fig. 8 and S16–S19 in the ESI† file). A conspicuous increase was observed in the absorbance intensity maxima which correspond to iodine, namely, at ∼227 nm (due to I_2_), ∼290 nm and ∼357 nm (due to polyiodide ions), which confirms the release of the adsorbate from CCP1–5 samples under ambient conditions. It is noteworthy that the amount of iodine released from CCP1–5 reached equilibrium after 45 minutes with a clear change in the color intensity of the solution from colorless to yellow (Fig. 8). These experimental observations strongly suggest that copolymers CCP1–5 can be employed as efficient iodine sorbents and can be easily regenerated either by simple heating or soaking in ethanol, therefore, rendering the recycling process very practical.

## 6. Dye adsorption

**Fig. 9:**
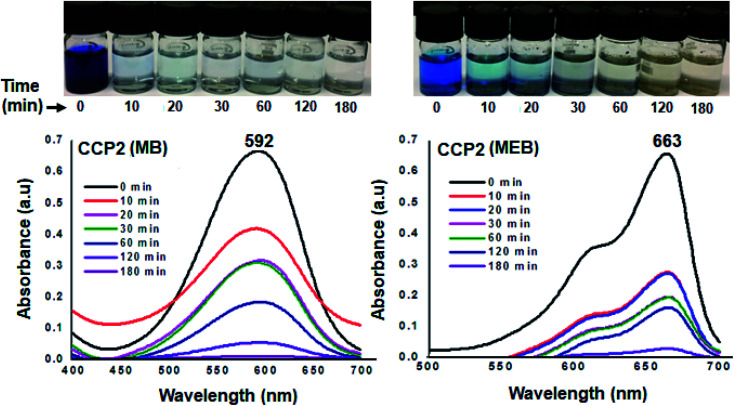
UV-Vis absorption spectra of aqueous solution MB (left) and MEB (right) in the presence of CCP2 at various time intervals (inset: photographs showing the color change upon dye adsorption).

The efficiency of the clathrochelate-based copolymers CCP1–5 to adsorb dyes from aqueous solution was carried out using methyl blue (MB, anionic dye) and methylene blue (MEB, cationic dye). The former is an important reagent for various biological and industrial applications whereas the latter is considered to be a primary source of pollution in water resources.^8,66–69^ The capacity of CCP1–5 to adsorb MB and MEB was investigated by stirring at ambient temperature a 4 mg sample of a target copolymer in a 5 mL of a 5 mg L^−1^ aqueous solution of either MB or MEB dyes. The adsorption tests were performed by recording the UV-visible absorbance spectra at different time intervals (Fig. 9 and S20–S27 in the ESI† file). Interestingly, all the clathrochelate-based copolymers CCP1–5 demonstrated an excellent adsorption capacity towards MB where more than 95% of the latter was removed within 2 h of reaction and a 100% adsorption was detected after 3 h of reaction. On the other hand, target copolymers CCP1–5 revealed a less efficient adsorption capacity towards MEB except for CCP2 which disclosed the adsorption of ∼70% of the dye in 10 minutes and ∼100% after 3 h (Fig. 9). Target copolymers CCP1,3–5 adsorbed only ∼70–80% of MEB even after long reaction times (Fig. S20–S27 in the ESI† file).

## 7. Conclusion

Five metalorganic copolymers CCP1–5 were prepared in very good yields *via* a one-step Sonogashira cross-coupling reaction between various dibrominated iron(ii) clathrochelate units with 1,4-diethynyl triptycene and 2,7-diethynyl-9,9-dimethyl fluorene synthons. Gel permeation chromatography of the soluble target copolymer CCP3 disclosed weight average molar mass *M*_w_ of ∼14 kDa and number average molar mass, Mn of ∼7 kDa. Porosity investigation divulged BET surface areas ranging from 45 to 337 m^2^ g^−1^ with greater porosity values for the fluorinated copolymers CCP5 bearing fluorene derivatives and CCP2 containing triptycene units, which disclosed surface areas of 279 m^2^ g^−1^ for the former copolymer and 337 m^2^ g^−1^ for the latter. Likewise, iodine uptake studies depicted adsorption capacities up to 200 wt% for the fluorinated copolymers CCP2,5. Furthermore, CCP1–5 demonstrate efficient adsorption of both methyl blue and methylene blue from aqueous solution with a higher preference towards the former. It is worth mentioning that the metalorganic copolymers CCP1–5 present several advantages, among others, their ease of synthesis and isolation, superior stability, in addition to the possibility for their utilization in the treatment of emission of toxic iodine vapors and organic dyes.

## Conflicts of interest

There are no conflicts to declare.

## Supplementary Material

RA-011-D1RA02357H-s001
