# Supplementary material for: Fluorinated Iron(ii) clathrochelate units in metalorganic based copolymers: improved porosity, iodine uptake, and dye adsorption properties
Source: RSC Adv. 2021 Apr 21;11(25):14986–95. doi: 10.1039/d1ra02357h (PMC8697800; doi:10.1039/d1ra02357h)
Supplement: RA-011-D1RA02357H-s001 [file RA-011-D1RA02357H-s001.pdf]

Electronic Supplementary Material (ESI) for RSC Advances.
This journal is © The Royal Society of Chemistry 2021

Supplementary Information

Fluorinated Iron(II) Clathrochelate Units in Metalorganic Based Copolymers: Improved Porosity, Iodine Uptake, and Dye Adsorption Properties

Suchetha Shetty,[a,b] Noorullah Baig,[a,b] Atikur Hassan[c] Saleh Al-Mousawi,[d] Neeladri Das,[c] and Bassam Alameddine*[a,b]

[a]Department of Mathematics and Natural Sciences, Gulf University for Science and Technology, Kuwait.

[b] Functional Materials Group – CAMB, GUST, Kuwait.

[c]Department of Chemistry, Indian Institute of Technology Patna, Patna 801106, Bihar, India.

[d]Department of Chemistry, University of Kuwait, Kuwait.

Contents

^{1}H NMR spectra of **CC2** and **CC3**	Figure S1, S2
^{13}C NMR spectra of **CC2**, **CC3** and **CCP3**	Figure S3-S5
EI-HRMS spectrum of **CC2**	Figure S6
EI-HRMS spectrum of **CC3**	Figure S7
FTIR spectra of **CCP1,2,4,5**	Figure S8-S11
High-resolution XPS spectra of **CCP1,2,4,5**	Figure S12-S15
UV-Vis absorption spectra of I2@**CCP1,3-5** in ethanol	Figure S16-S19
UV-Vis absorption spectra of MB & MEB in **CCP1,3-5**	Figure S20-S27

*Corresponding author. *E-mail address*: alameddine.b@gust.edu.kw

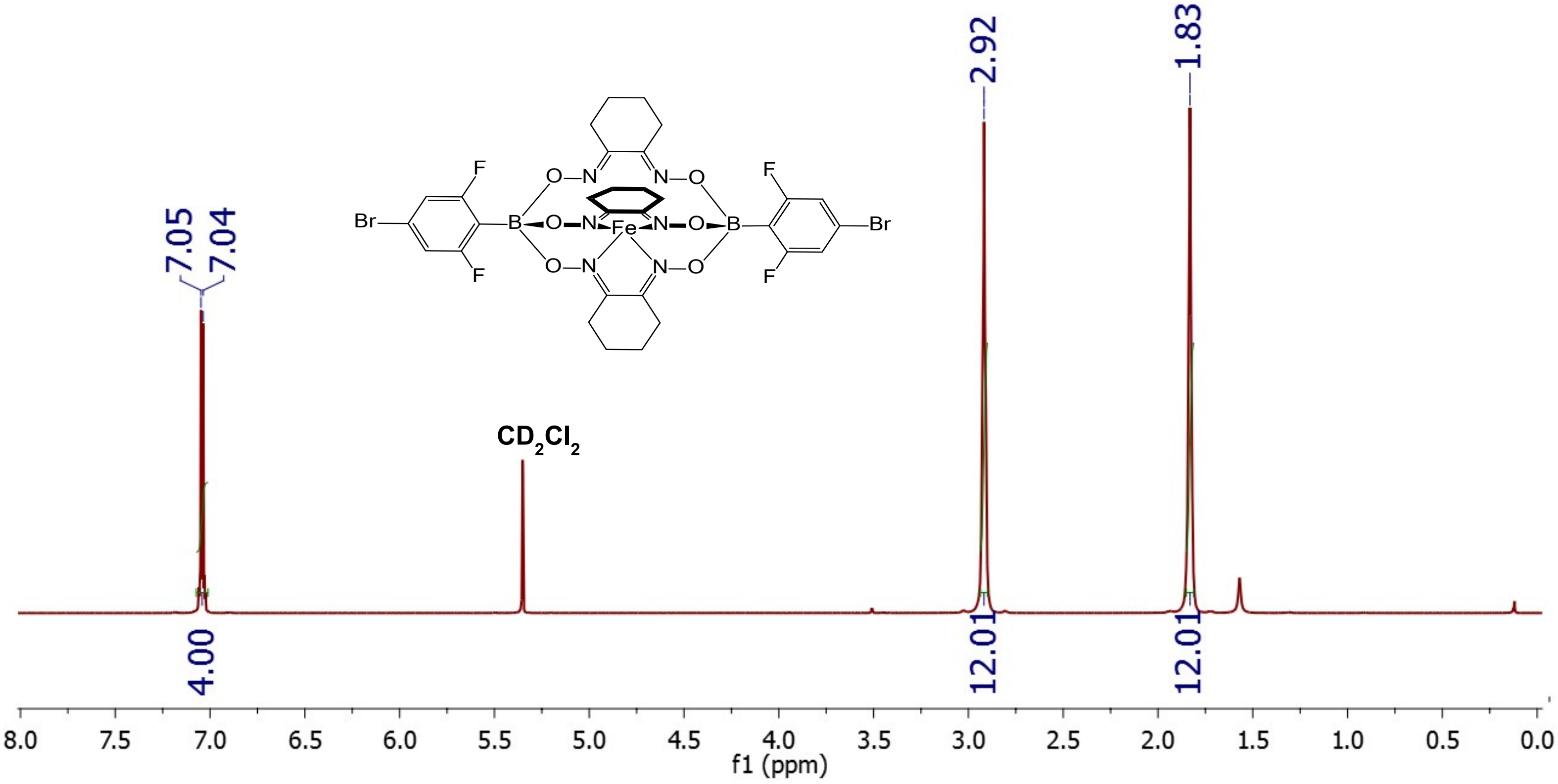

Fig. S1. ^{1}H NMR spectrum of **CC2** recorded in CD_2Cl_2

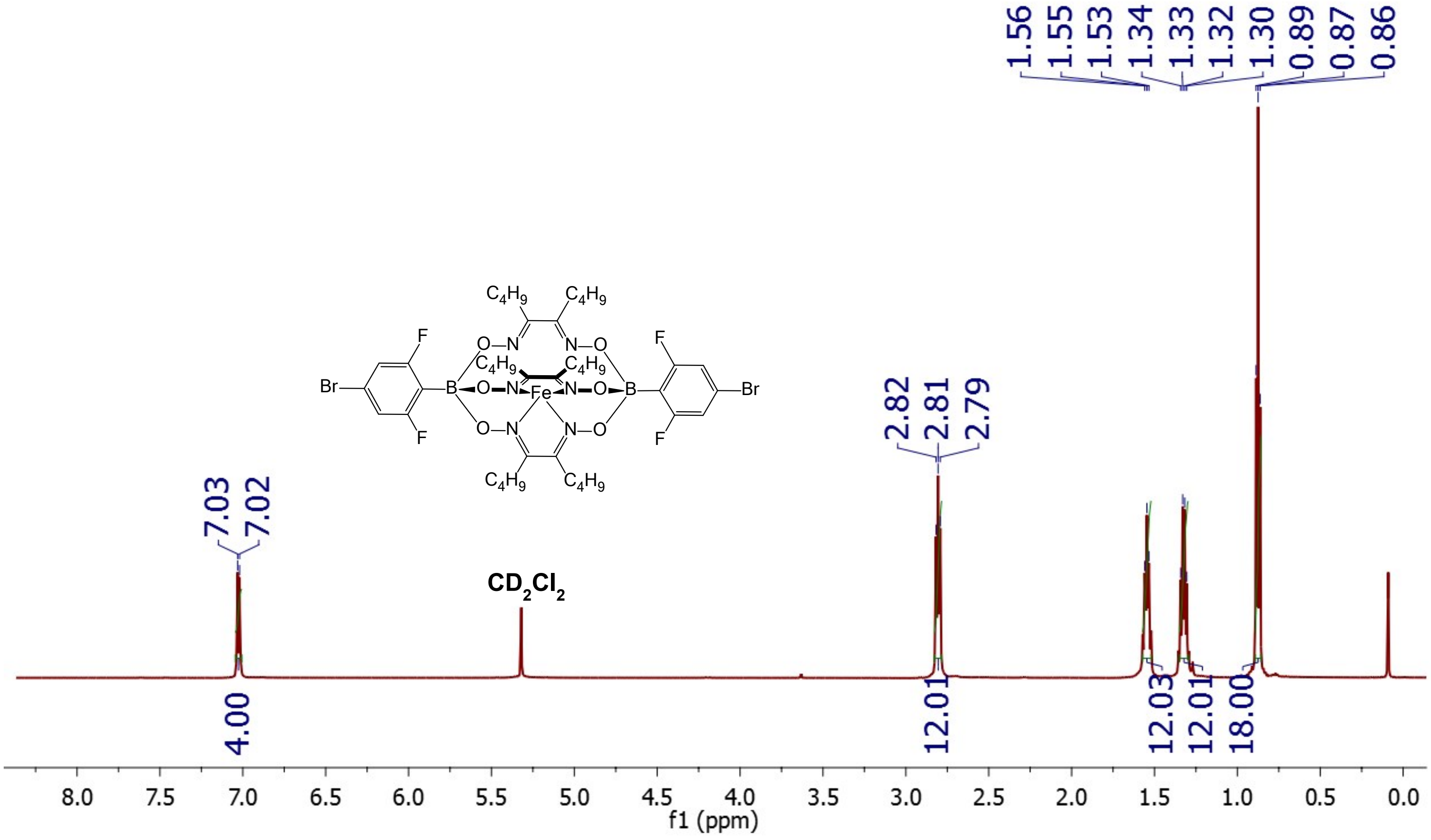

Fig. S2. ^{1}H NMR spectrum of **CC3** recorded in CD_2Cl_2

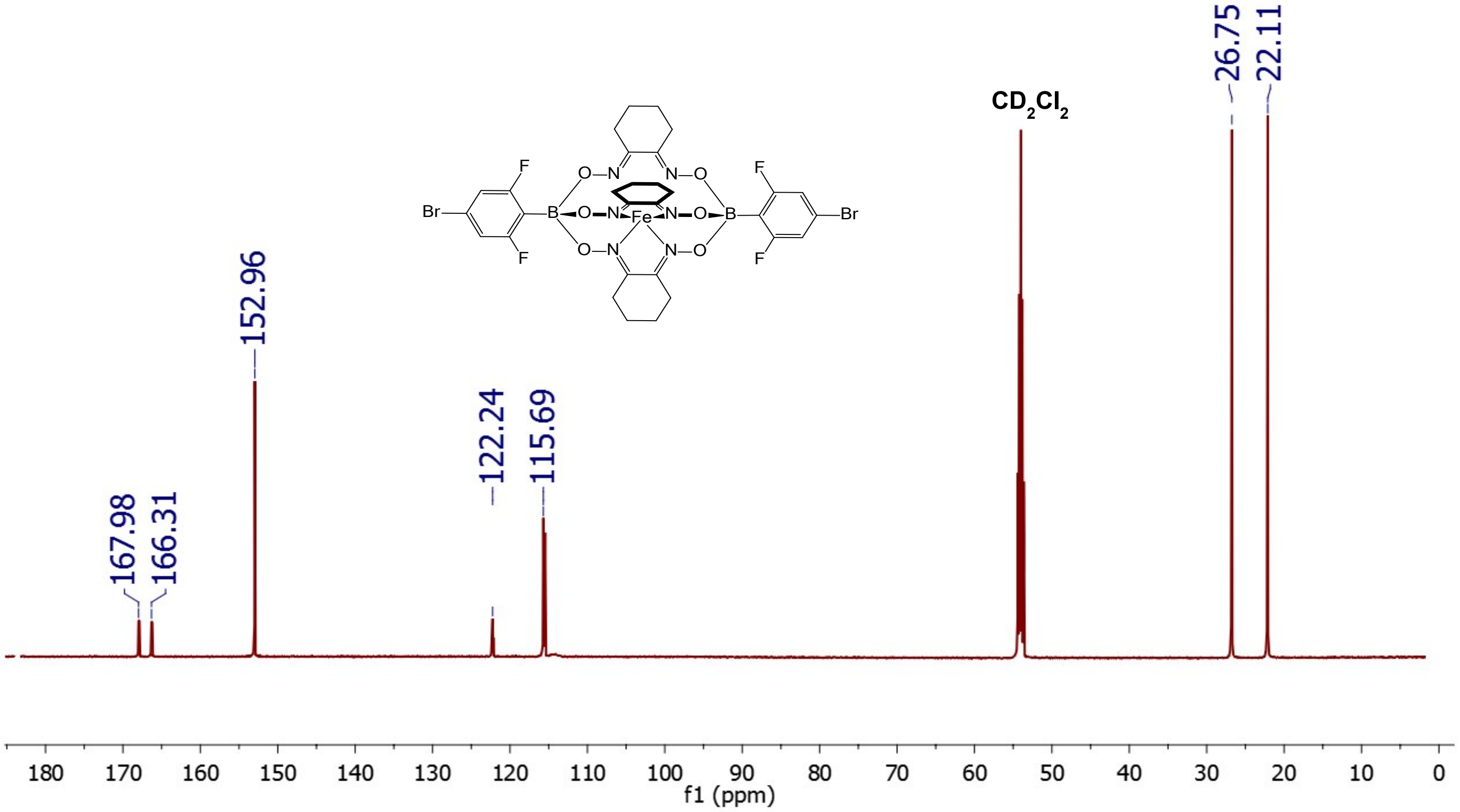

Fig. S3. ^{13}C NMR spectrum of **CC2** recorded in CD_2Cl_2

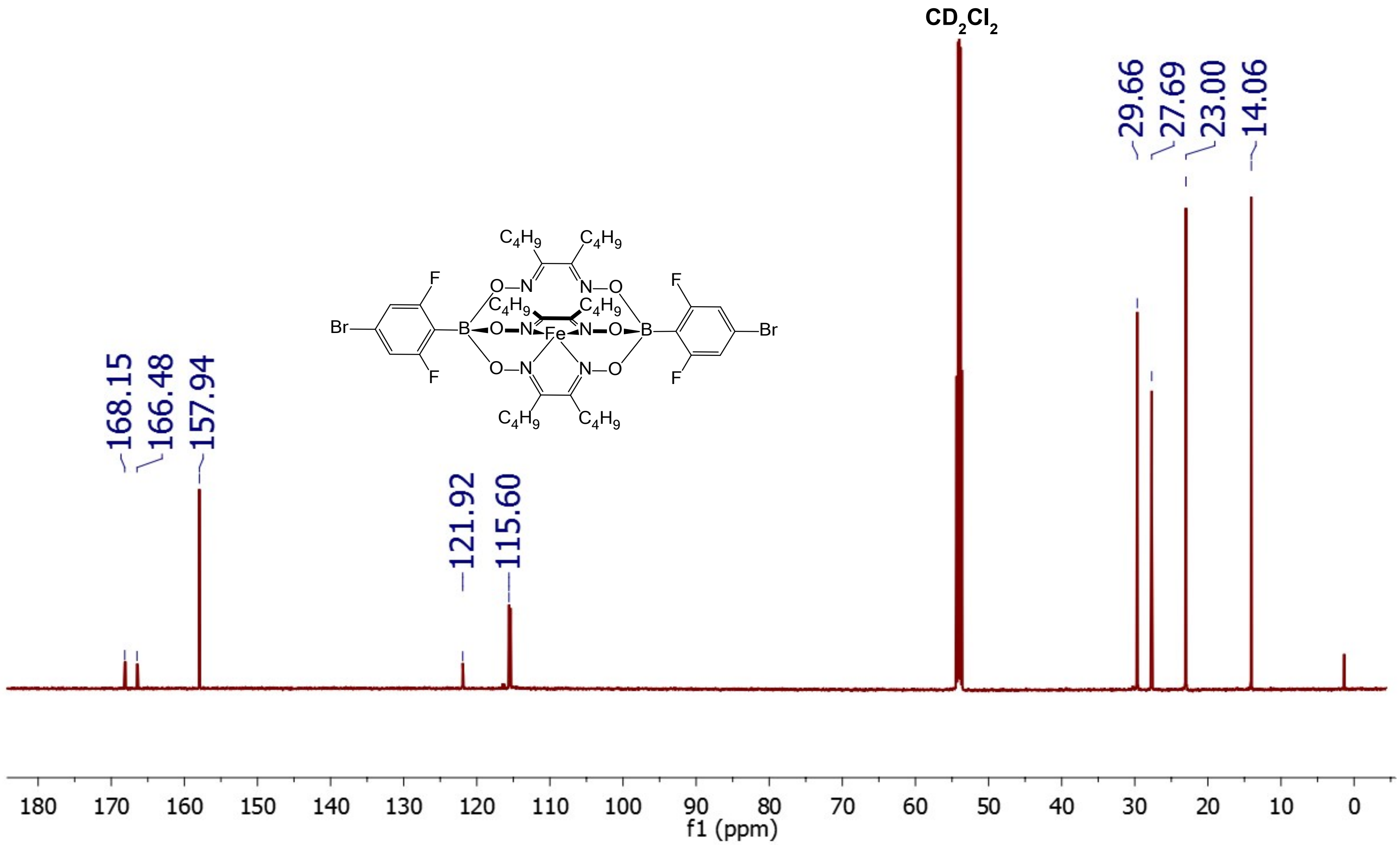

Fig. S4. ^{13}C NMR spectrum of **CC3** recorded in CD_2Cl_2

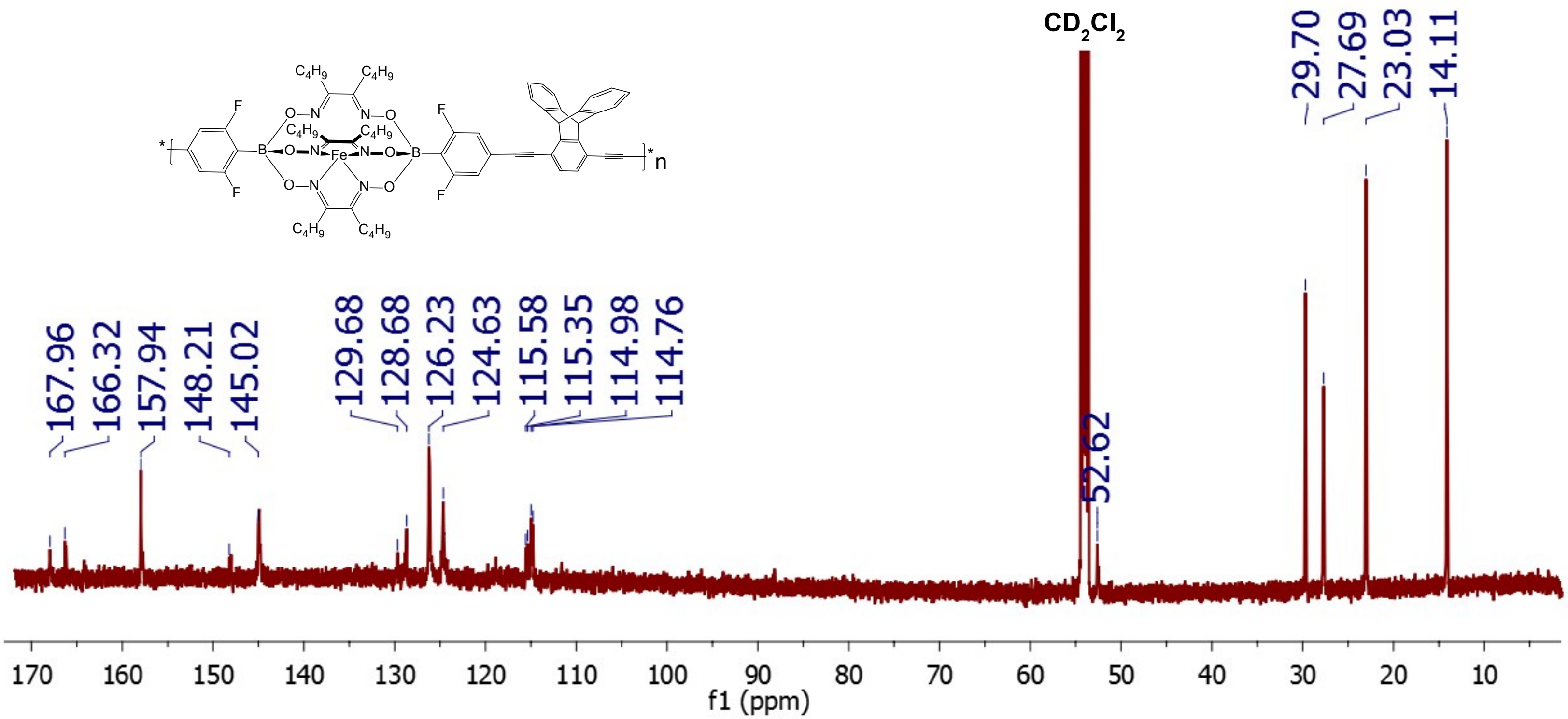

Fig. S5. ^{13}C NMR spectrum of **CCP3** recorded in CD_2Cl_2

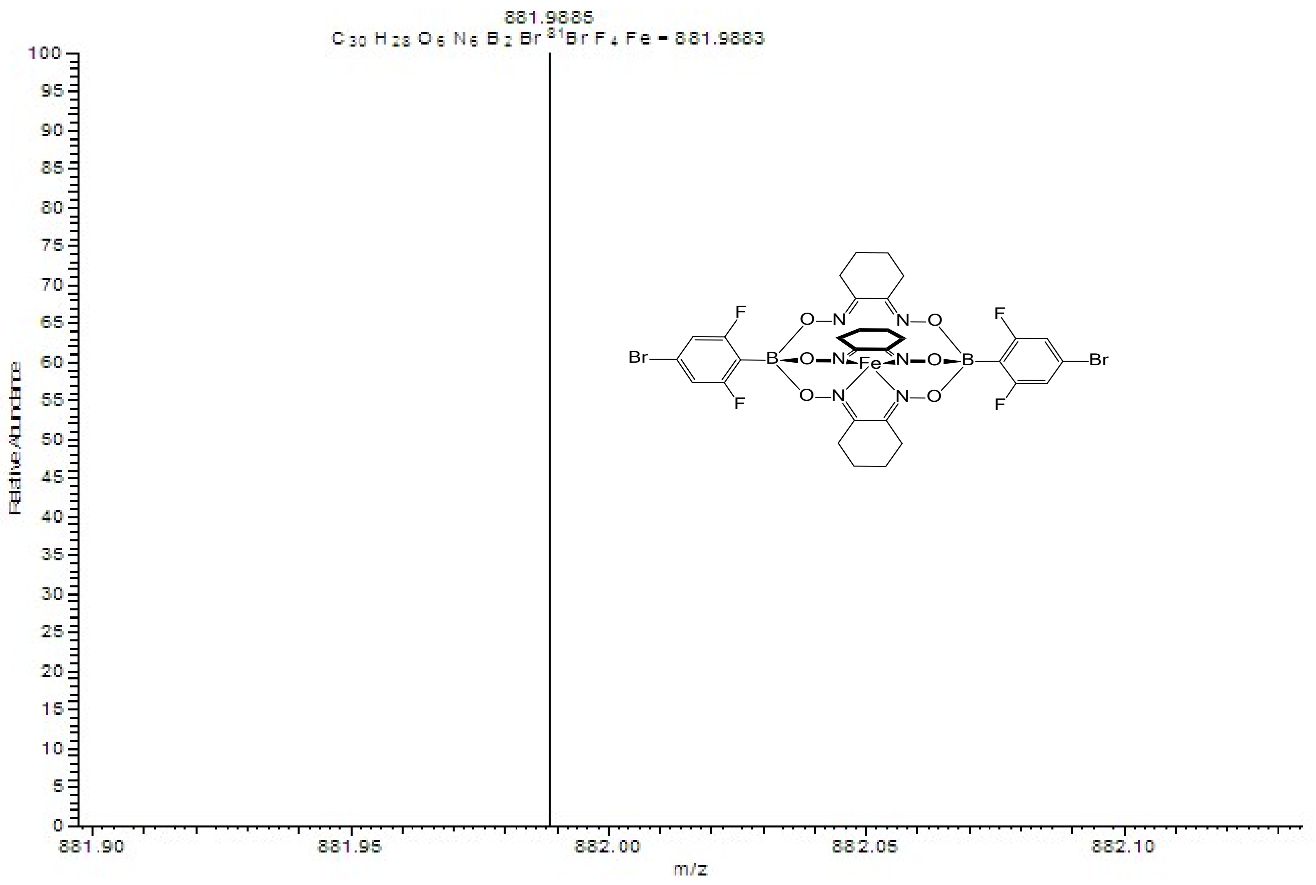

Fig. S6. EI-HRMS spectrum of **CC2**

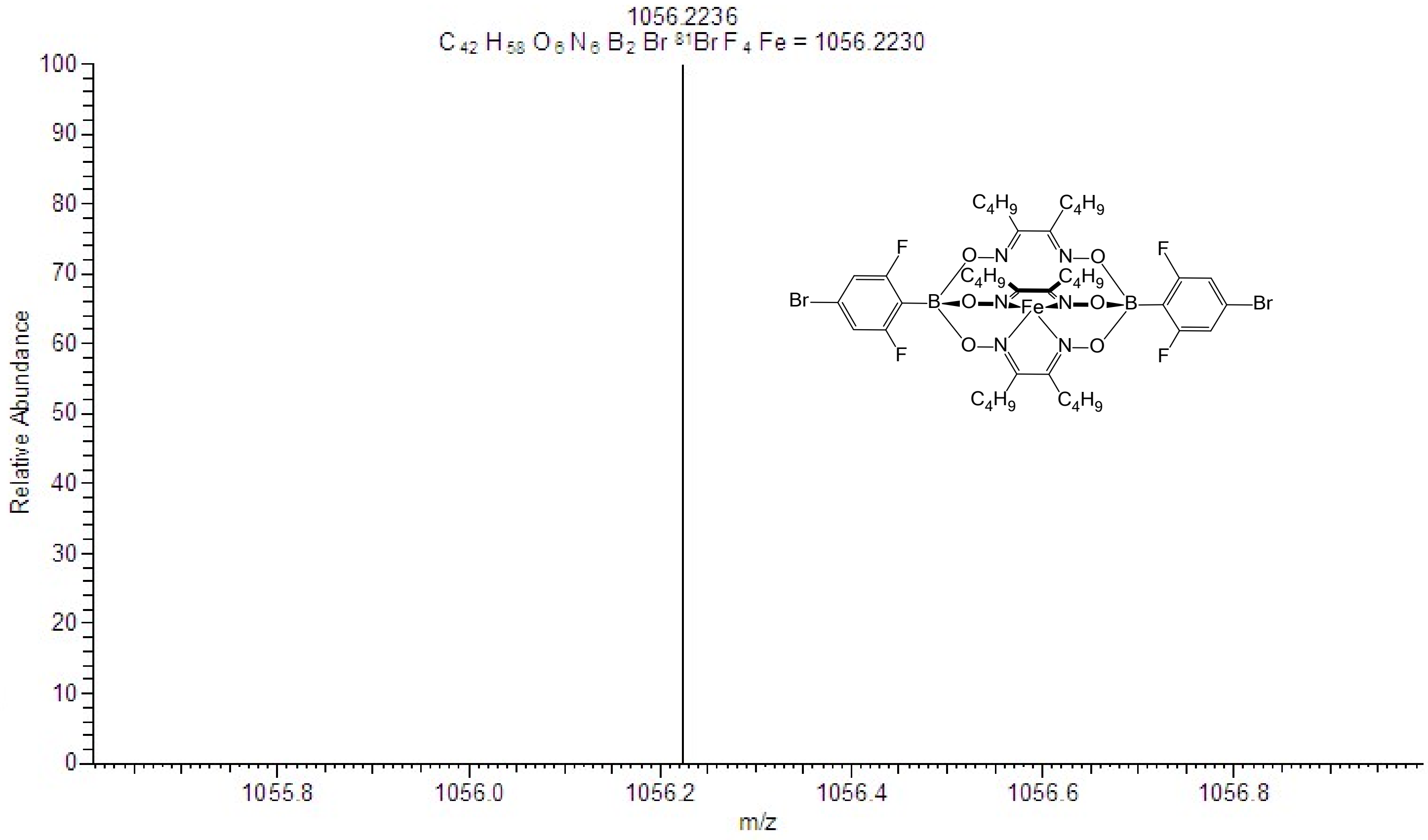

Fig. S7. EI-HRMS spectrum of **CC3**

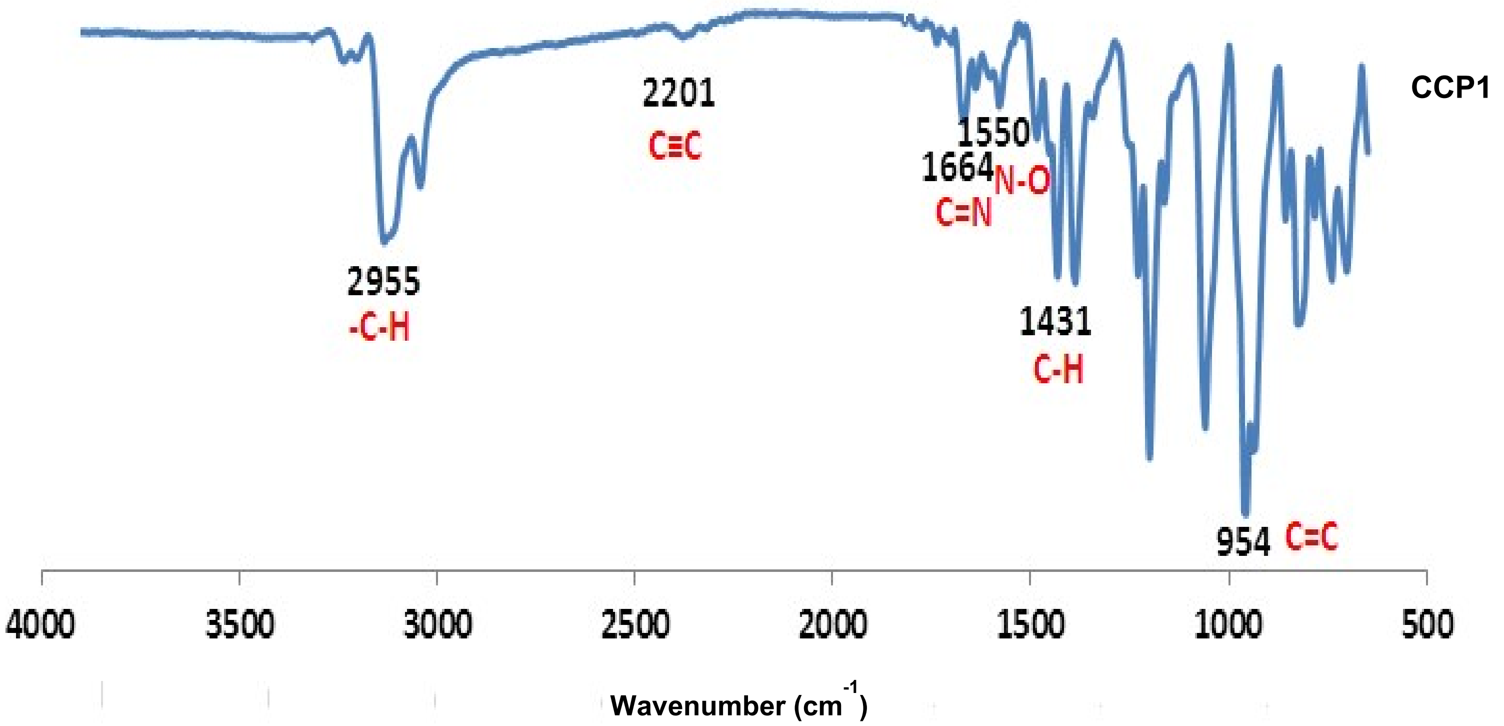

Fig. S8. FTIR spectrum of **CCP1**

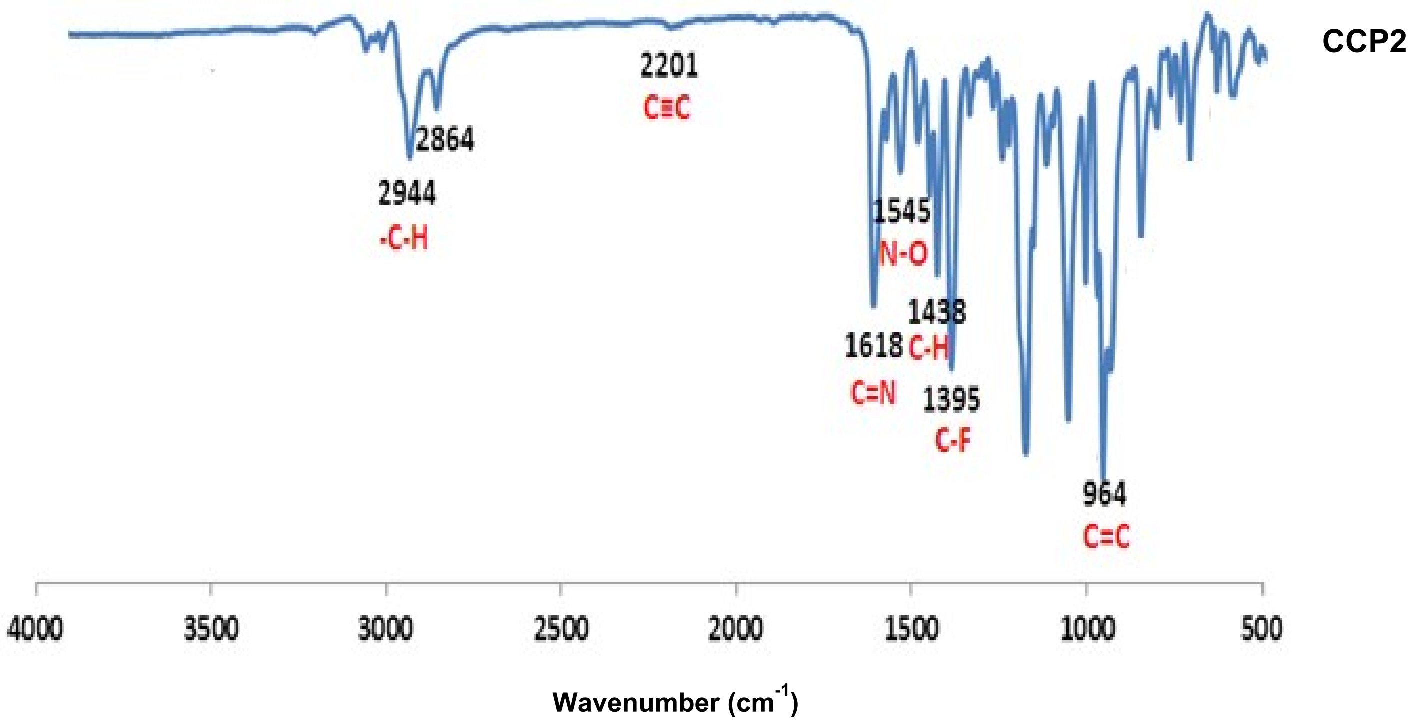

Fig. S9. FTIR spectrum of **CCP2**

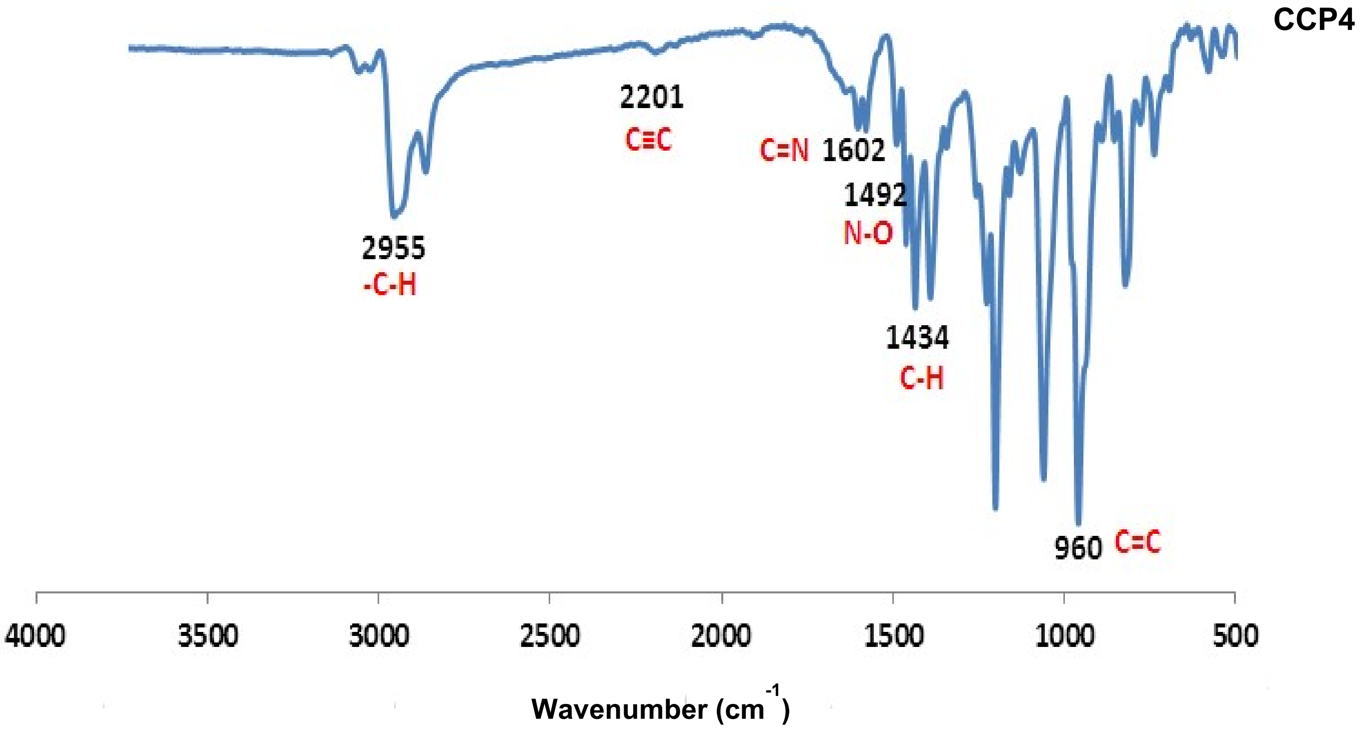

Fig. S10. FTIR spectrum of **CCP4**

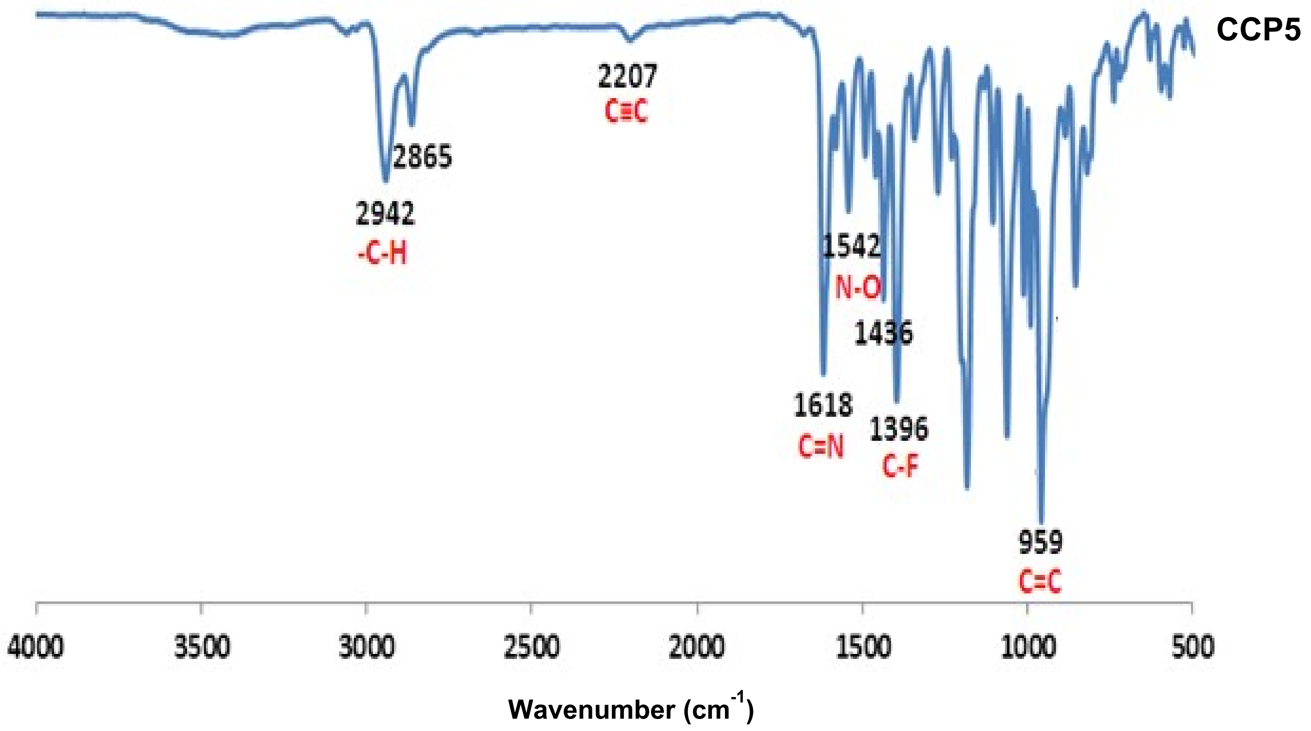

Fig. S11. FTIR spectrum **CCP5**

Name	Peak BE
C1s	284.61
C1s A	285.29
C1s B	285.96
O1s	532.23
O1s A	532.20
N1s	400.96
N1s A	401.56
$Fe2p_{3/2}$	709.13
$Fe2p_{1/2}$	722.57
B1s	190.96

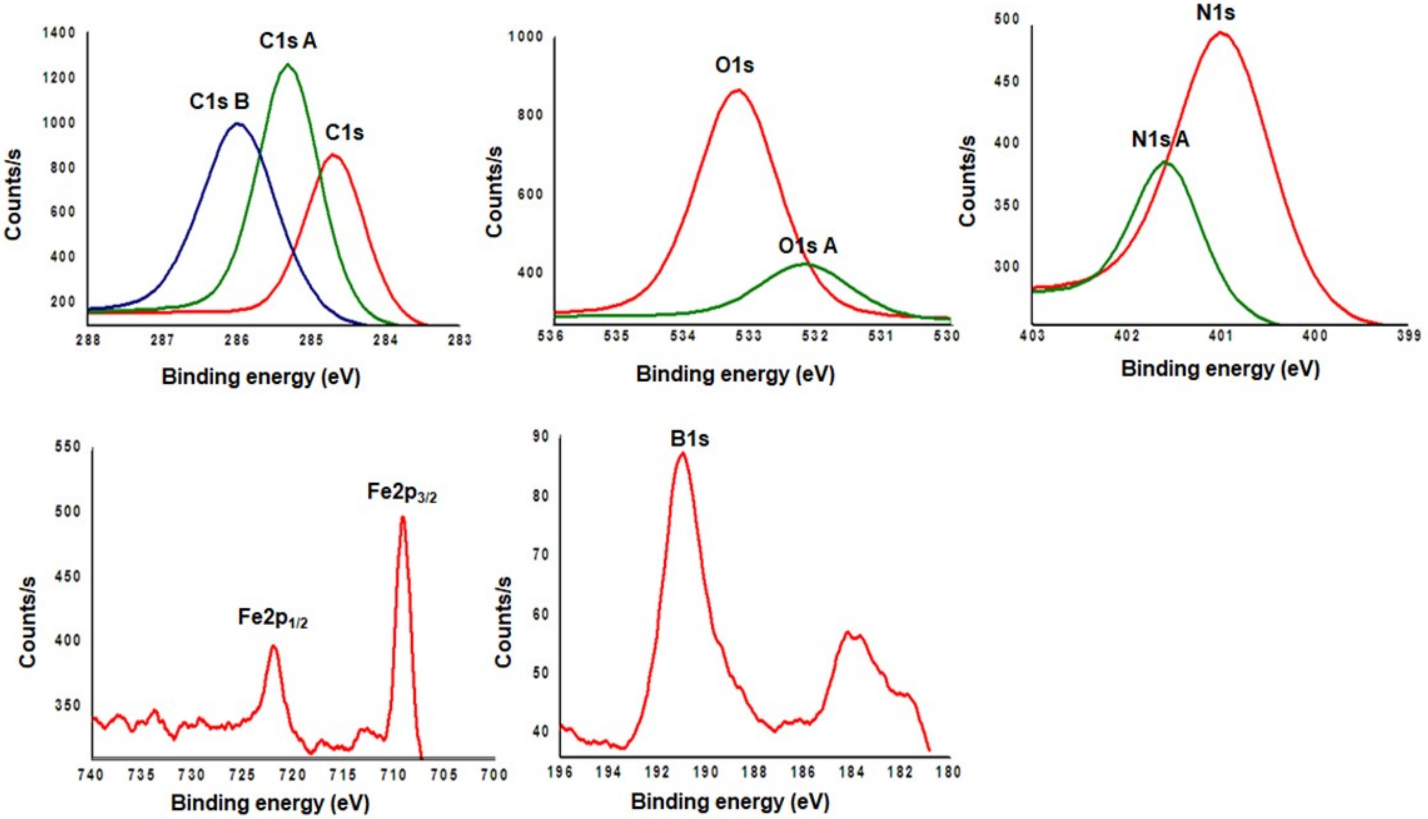

Fig. S12. High-resolution XPS spectra of C1s, O1s, N1s, Fe2p and B1s of **CCP1.**

Name	Peak BE
C1s	284.64
C1s A	285.30
C1s B	285.96
C1s C	291.37
O1s	532.96
O1s A	532.22
N1s	400.61
Fe2p$_{3/2}$	709.23
Fep$_{1/2}$	722.55
F1s	686.63
B1s	191.21

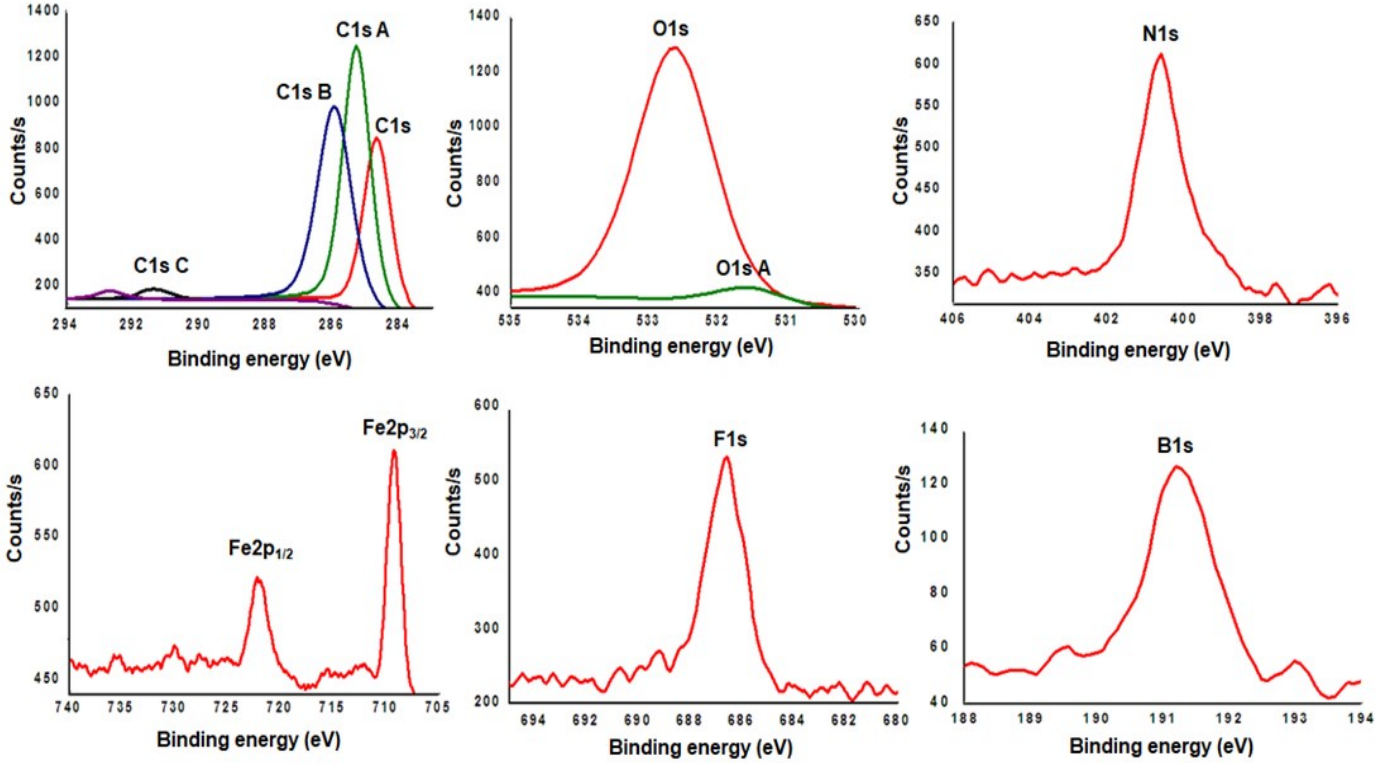

Fig. S13. High-resolution XPS spectra of C1s, O1s, N1s, Fe2p, F1s and B1s of **CCP2**.

Name	Peak BE
C1s	284.59
C1s A	285.31
C1s B	285.99
O1s	532.85
O1s A	532.25
N1s	400.61
$Fe2p_{3/2}$	709.31
$Fe2p_{1/2}$	722.55
B1s	191.25

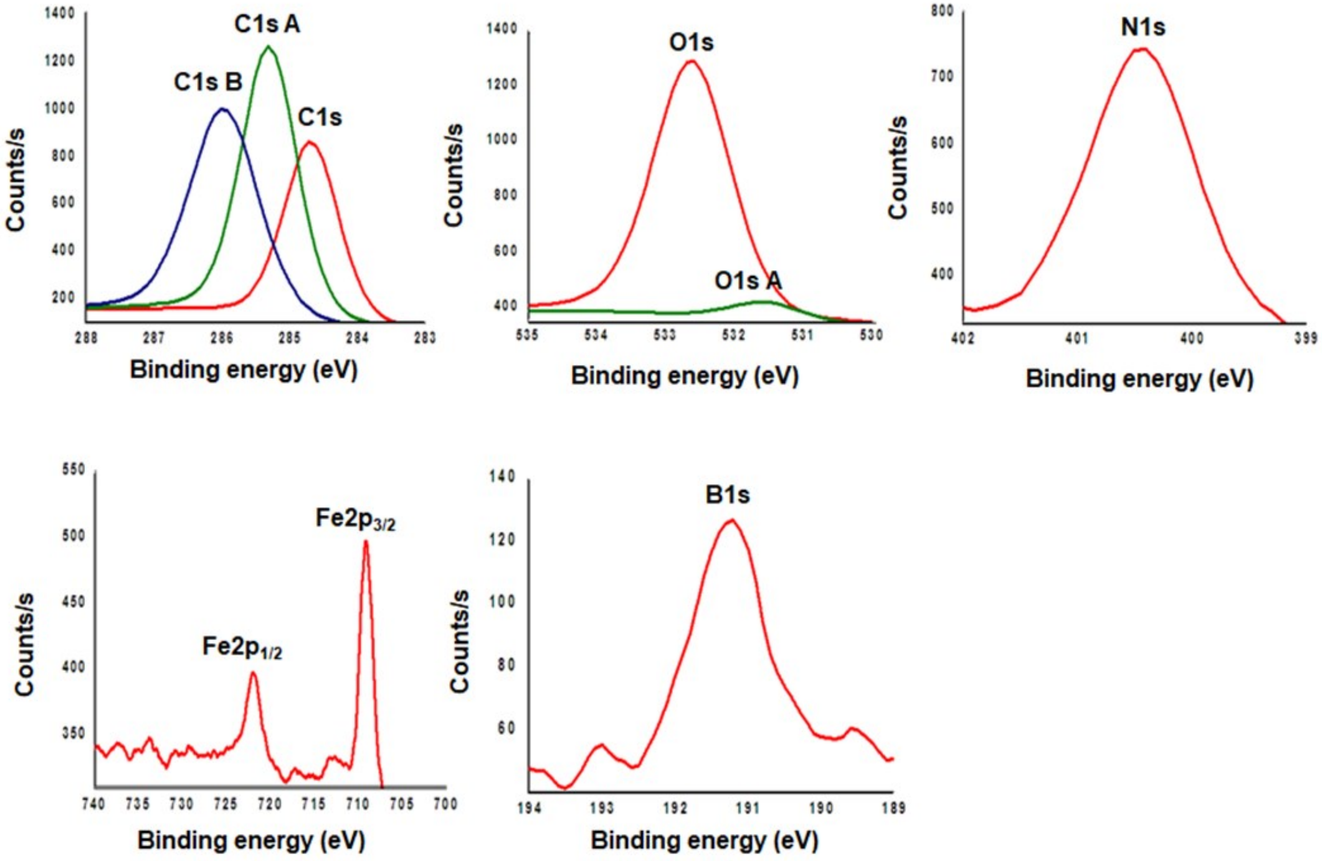

Fig. S14. High-resolution XPS spectra of C1s, O1s, N1s, Fe2p and B1s of **CCP4**

Name	Peak BE
C1s	284.67
C1s A	285.27
C1s B	287.01
O1s	532.40
O1s A	531.57
N1s	400.42
$Fe2p_{3/2}$	709.27
$Fe2p_{1/2}$	722.01
F1s	686.72
B1s	191.23

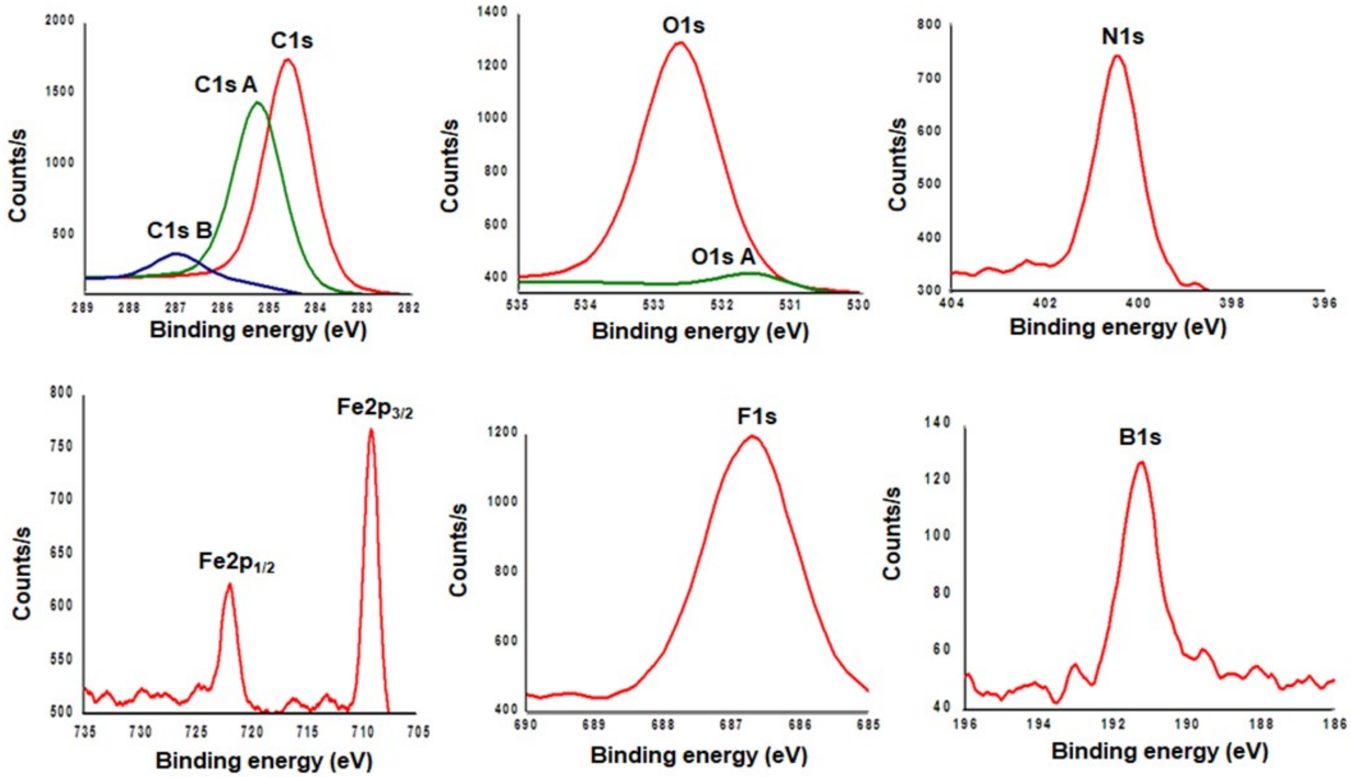

Fig. S15. High-resolution XPS spectra of C1s, O1s, N1s, Fe2p, F1s and B1s of **CCP5**

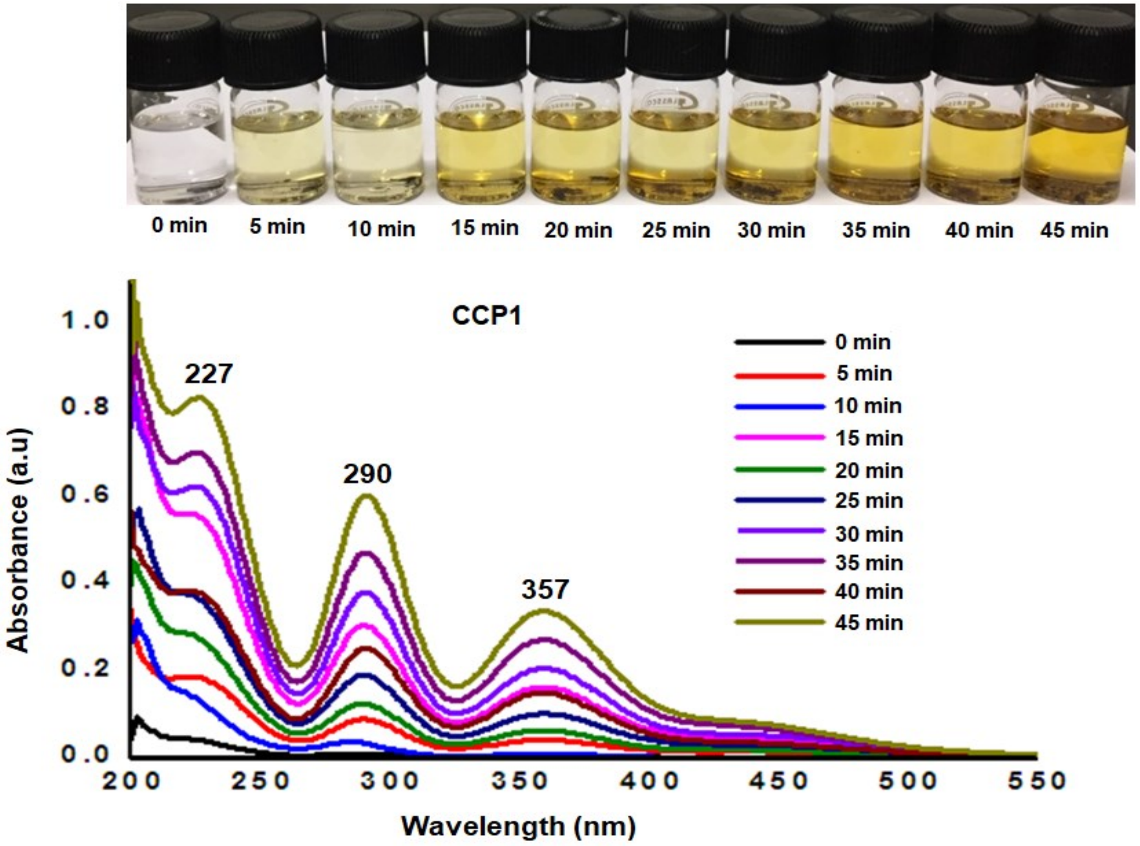

Fig. S16. UV-Vis absorption spectra upon immersion of I2@**CCP1** in ethanol.

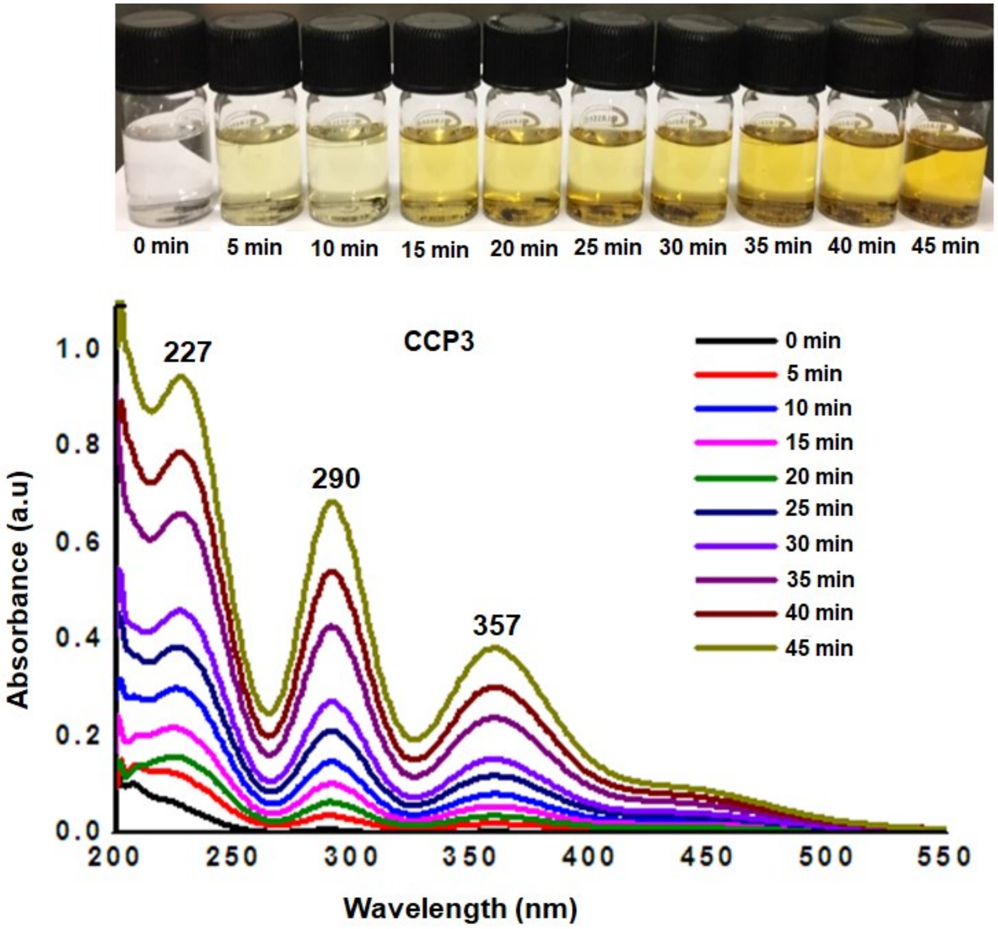

Fig. S17. UV-Vis absorption spectra upon immersion of I2@**CCP3** in ethanol.

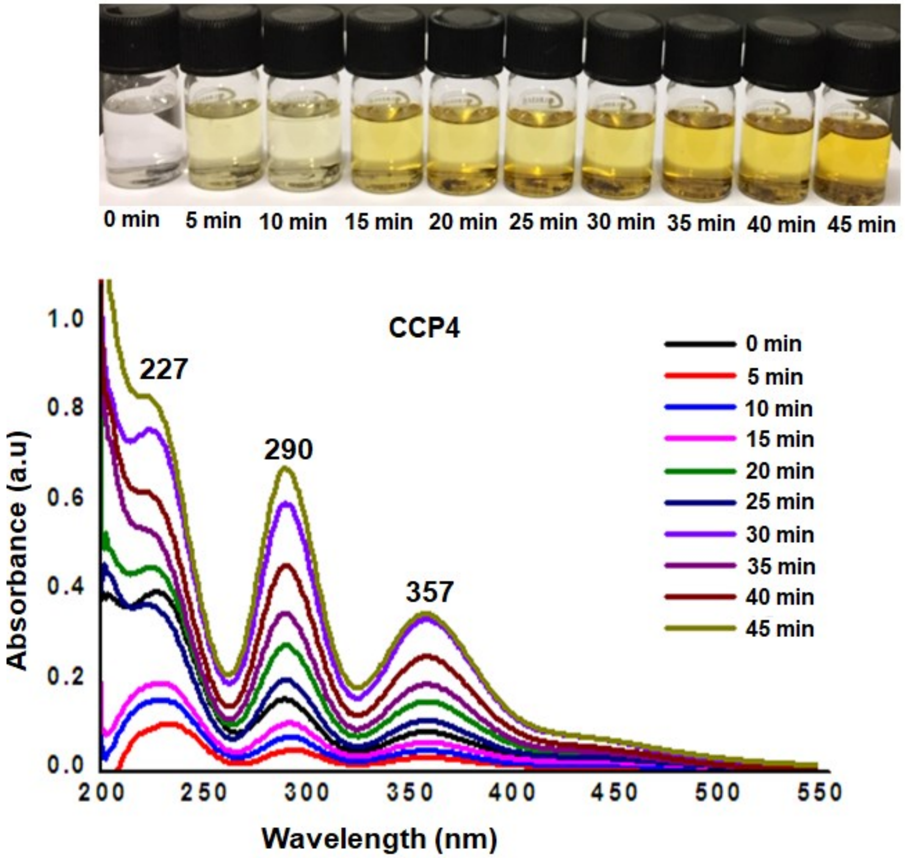

Fig. S18. UV-Vis absorption spectra upon immersion of I2@**CCP4** in ethanol

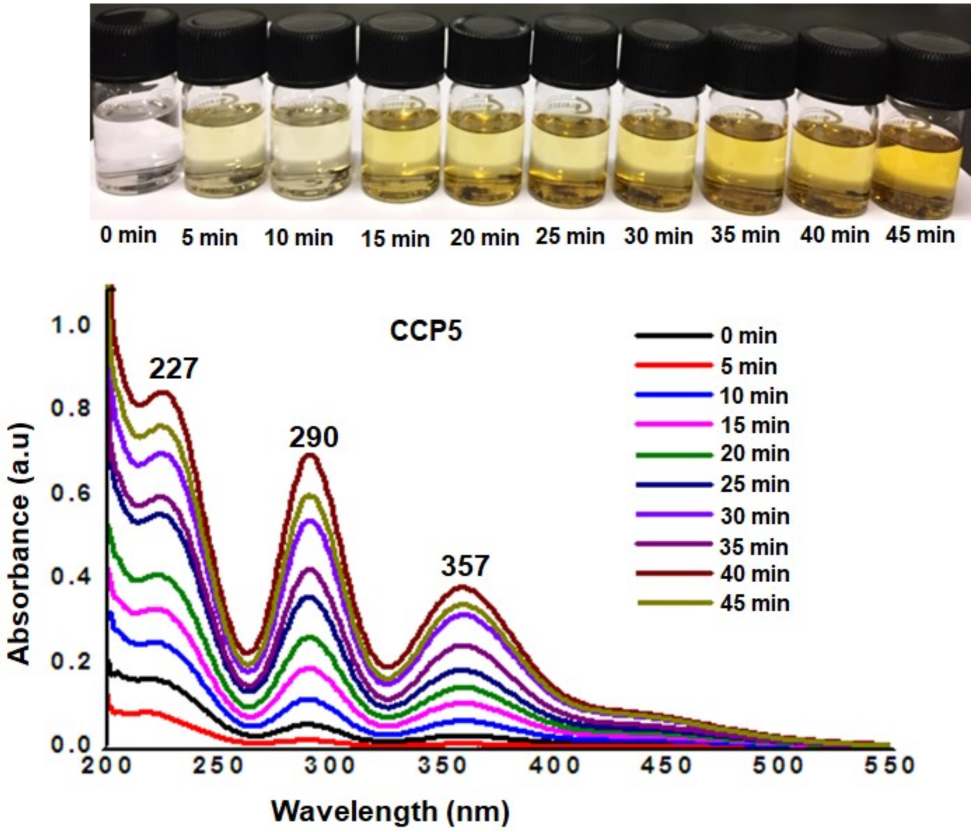

Fig. S19. UV-Vis absorption spectra upon immersion of I2@**CCP5** in ethanol

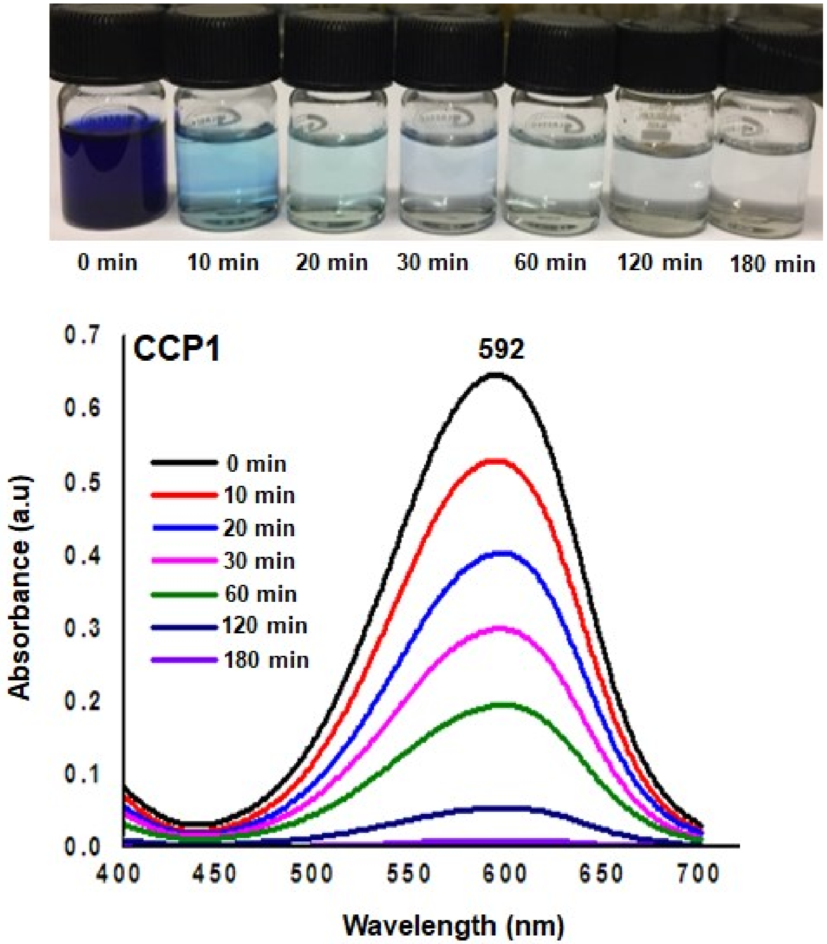

Fig. S20. UV-Vis absorption spectrum of aqueous solution MB in the presence of **CCP1** at various time intervals

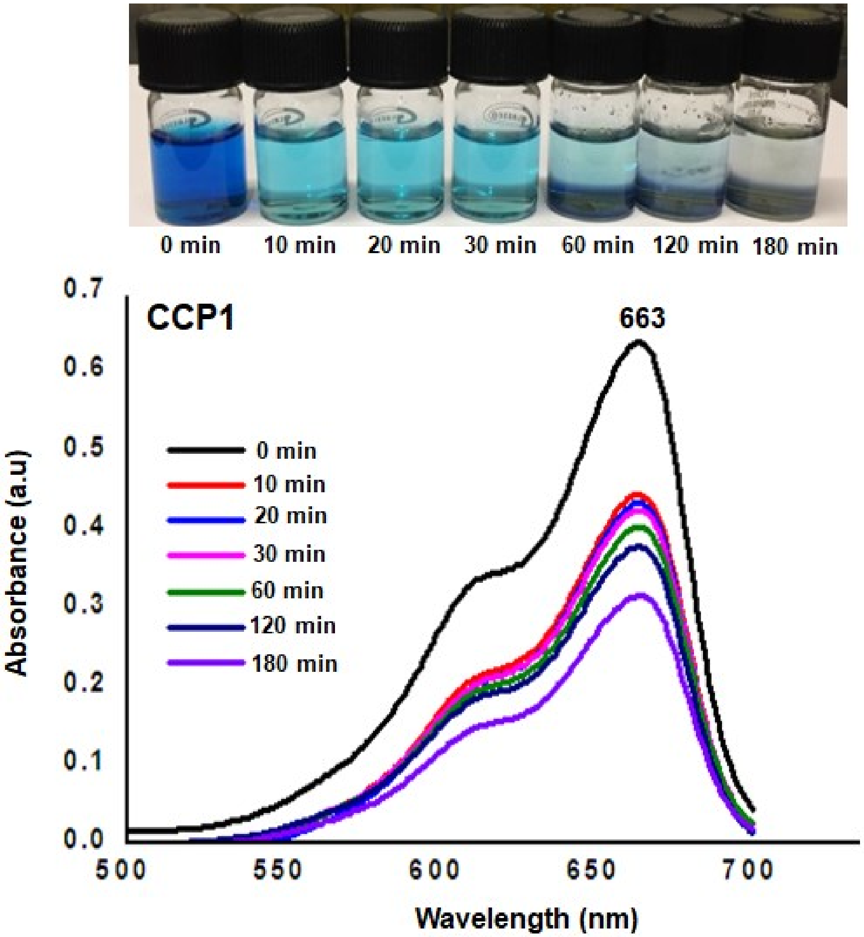

Fig. S21. UV-Vis absorption spectrum of aqueous solution MEB in the presence of **CCP1** at various time intervals

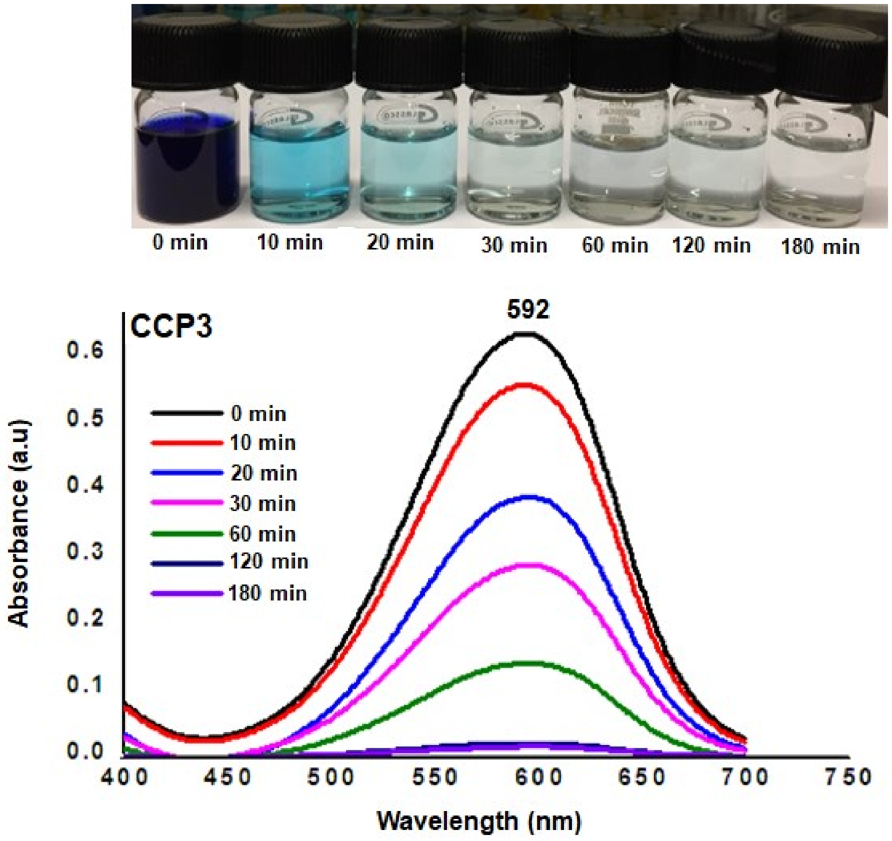

Fig. S22. UV-Vis absorption spectrum of aqueous solution MB in the presence of **CCP3** at various time intervals

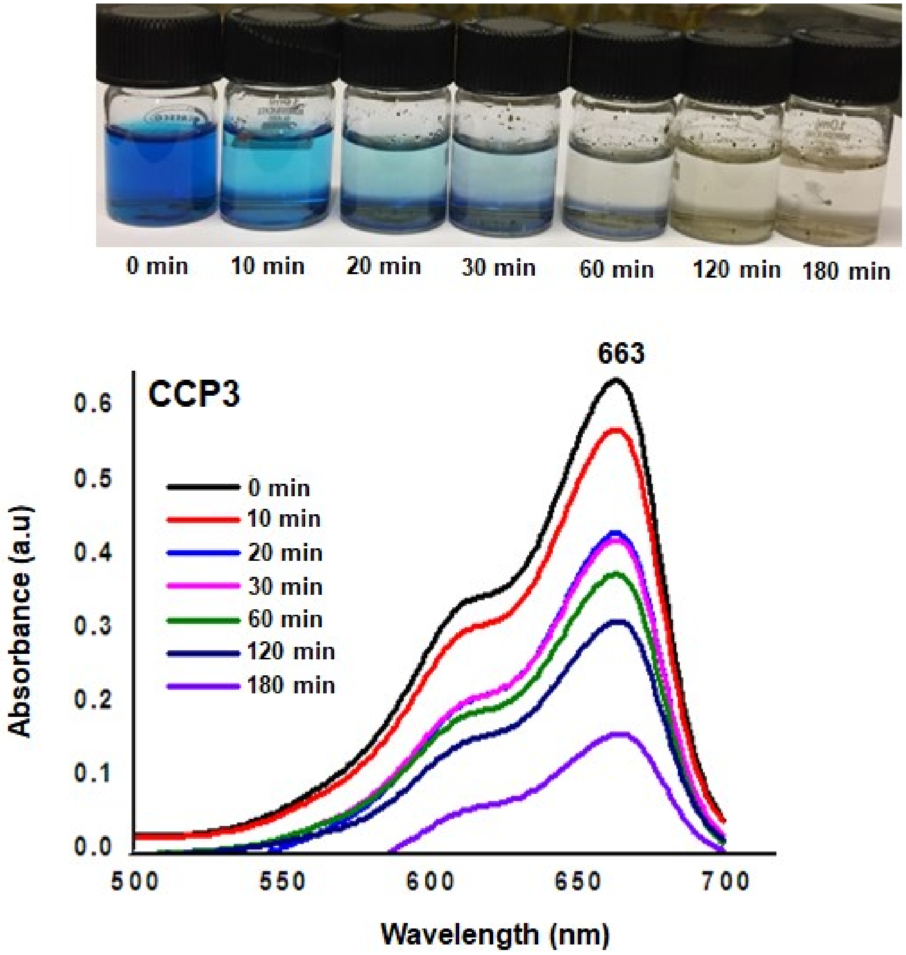

Fig. S23. UV-Vis absorption spectrum of aqueous solution MEB in the presence of **CCP3** at various time intervals

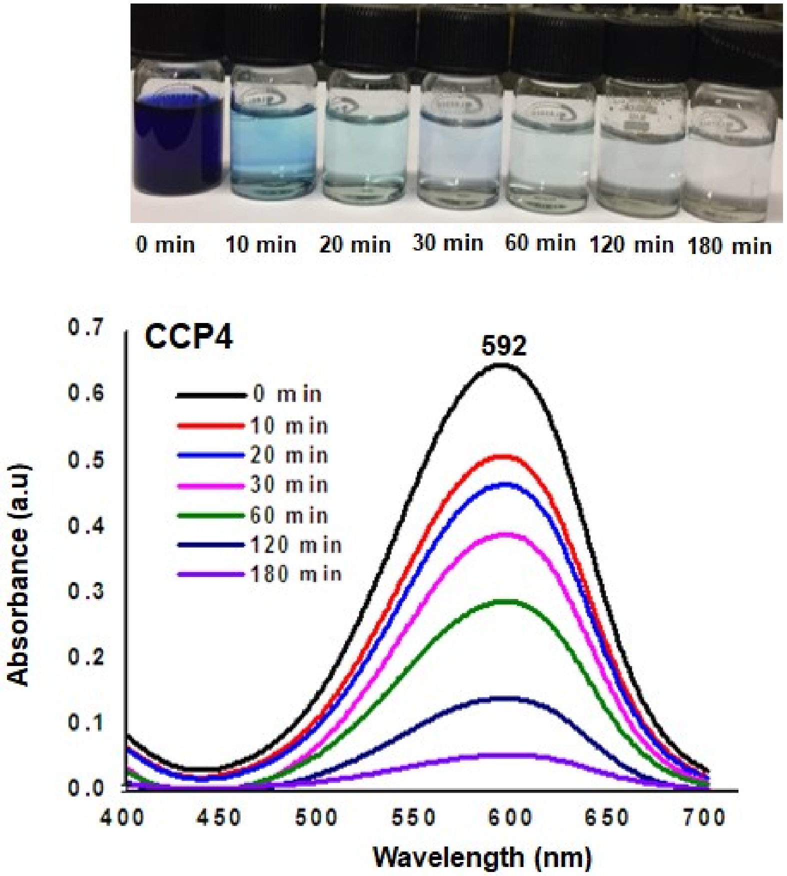

Fig. S24. UV-Vis absorption spectrum of aqueous solution MB in the presence of **CCP4** at various time intervals

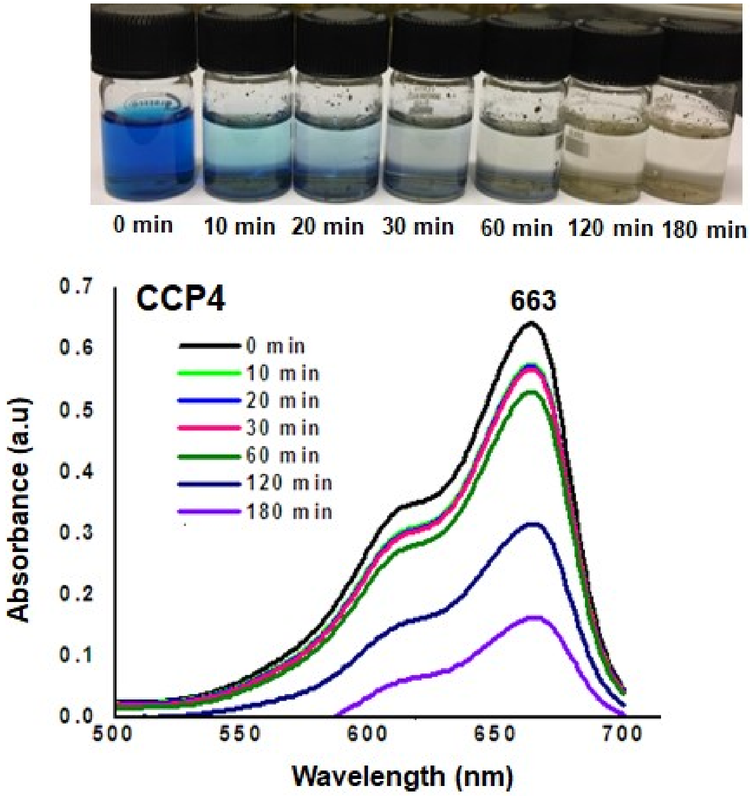

Fig. S25. UV-Vis absorption spectrum of aqueous solution MEB in the presence of **CCP4** at various time intervals

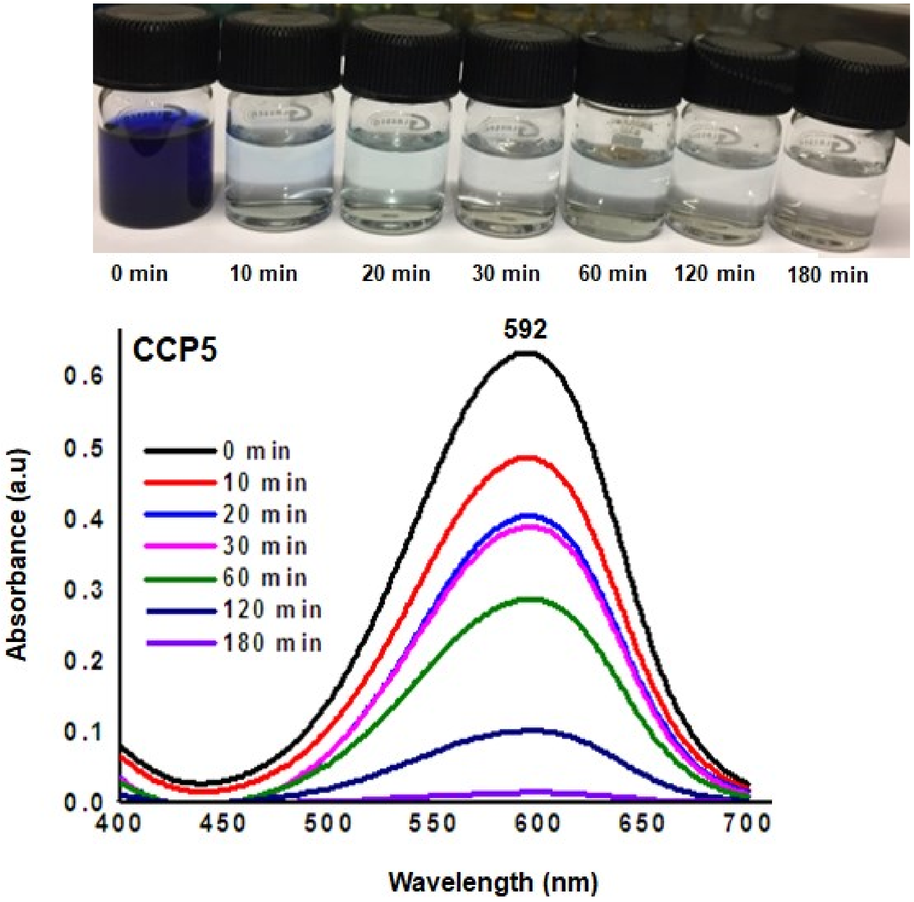

Fig. S26. UV-Vis absorption spectrum of aqueous solution MB in the presence of **CCP5** at various time intervals

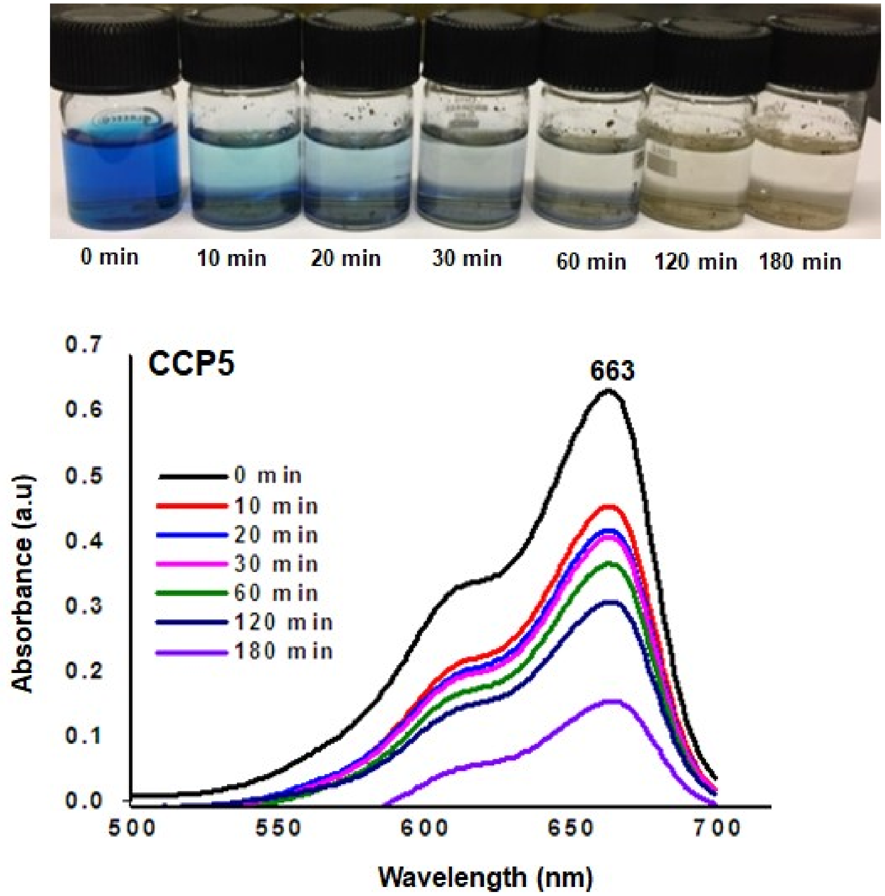

Fig. S27. UV-Vis absorption spectrum of aqueous solution MEB in the presence of **CCP5** at various time intervals
